# Natural history and genetic study of *LAMA2*-related muscular dystrophy in a large Chinese cohort

**DOI:** 10.1186/s13023-021-01950-x

**Published:** 2021-07-19

**Authors:** Dandan Tan, Lin Ge, Yanbin Fan, Xingzhi Chang, Shuang Wang, Cuijie Wei, Juan Ding, Aijie Liu, Shuo Wang, Xueying Li, Kai Gao, Haipo Yang, Chengli Que, Zhen Huang, Chunde Li, Ying Zhu, Bing Mao, Bo Jin, Ying Hua, Xiaoli Zhang, Bingbing Zhang, Wenhua Zhu, Cheng Zhang, Yanjuan Wang, Yun Yuan, Yuwu Jiang, Anne Rutkowski, Carsten G. Bönnemann, Xiru Wu, Hui Xiong

**Affiliations:** 1 Department of Pediatrics, Peking University First Hospital, Beijing, 100034 China; 2 Department of Statistics, Peking University First Hospital, Beijing, 100034 China; 3 Department of Respiratory and Critical Care Medicine, Peking University First Hospital, Beijing, 100034 China; 4 Department of Rehabilitation Medicine, Peking University First Hospital, Beijing, 100034 China; 5 Department of Orthopedic/Spine Surgery, Peking University First Hospital, Beijing, 100034 China; 6 Department of Radiology, Peking University First Hospital, Beijing, 100034 China; 7 Department of Neurology, Wuhan Children’s Hospital, Wuhan, 430015 Hubei Province China; 8 Department of Neurology, Children’s Hospital of Nanjing Medical University, Nanjing, 210008 Jiangsu Province China; 9 Department of Pediatrics, Wuxi Children’s Hospital, Wuxi, 214000 Jiangsu Province China; 10 Department of Pediatrics, The Third Affiliated Hospital of Zhengzhou University, Zhengzhou, 450052 Henan Province China; 11 Department of Neurology, Children’s Hospital of Soochow University, Suzhou, 215025 Jiangsu Province China; 12 Department of Neurology, Huashan Hospital, Fudan University, Shanghai, 200040 China; 13 Department of Neurology, The First Affiliated Hospital, Sun Yat-Sen University, Guangzhou, 510080 Guangdong Province China; 14 Department of Neurology, School of Medicine, Chengdu Women’s & Children’s Central Hospital, University of Electronic Science and Technology of China, Chengdu, 610091 Sichuan Province China; 15 Department of Neurology, Peking University First Hospital, Beijing, 100034 China; 16 Kaiser Permanente SCPMG Cure CMD, Los Angeles, CA USA; 17 Neuromuscular and Neurogenetic Disorders of Childhood Section, National Institute of Neurological Disorders and Stroke, National Institutes of Health, Bethesda, MD USA

**Keywords:** Natural history, Genotype, *LAMA2*, Muscular dystrophy, Rare diseases

## Abstract

**Background:**

*LAMA2*-related muscular dystrophy including *LAMA2*-related congenital muscular dystrophy (*LAMA2*-CMD) and autosomal recessive limb-girdle muscular dystrophy-23 (LGMDR23) is caused by *LAMA2* pathogenic variants. We aimed to describe the natural history and establish genotype–phenotype correlations in a large cohort of Chinese patients with *LAMA2*-related muscular dystrophy.

**Methods:**

Clinical and genetic data of *LAMA2*-related muscular dystrophy patients enrolled from ten research centers between January 2003 and March 2021 were collected and analyzed.

**Results:**

One hundred and thirty patients (116 *LAMA2*-CMD and 14 LGMDR23) were included. *LAMA2*-CMD group had earlier onset than LGMDR23 group. Head control, independent sitting and ambulation were achieved in 76.3%, 92.6% and 18.4% of *LAMA2*-CMD patients at median ages of 6.0 months (range 2.0–36.0 months), 11.0 months (range 6.0–36.0 months), and 27.0 months (range 18.0–84.0 months), respectively. All LGMDR23 patients achieved independent ambulation at median age of 18.0 months (range 13.0–20.0 months). Motor regression in *LAMA2*-CMD mainly occurred concurrently with rapid progression of contractures during 6–9 years old. Twenty-four *LAMA2*-related muscular dystrophy patients died, mostly due to severe pneumonia. Seizures occurred in 35.7% of LGMDR23 and 9.5% of *LAMA2*-CMD patients. Forty-six novel and 97 known *LAMA2* disease-causing variants were identified. The top three high-frequency disease-causing variants in Han Chinese patients were c.7147C > T (p.R2383*), exon 4 deletion, and c.5156_5159del (p.K1719Rfs*5). In *LAMA2*-CMD, splicing variants tended to be associated with a relatively mild phenotype. Nonsense variants were more frequent in *LAMA2*-CMD (56.9%, 66/116) than in LGMDR23 (21.4%, 3/14), while missense disease-causing variants were more frequent in LGMDR23 (71.4%, 10/14) than in *LAMA2*-CMD (12.9%, 15/116). Copy number variations were identified in 26.4% of survivors and 50.0% of nonsurvivors, suggesting that copy number variations were associated with lower rate of survival (*p* = 0.029).

**Conclusions:**

This study provides better understandings of natural history and genotype–phenotype correlations in *LAMA2*-related muscular dystrophy, and supports therapeutic targets for future researches.

**Supplementary Information:**

The online version contains supplementary material available at 10.1186/s13023-021-01950-x.

## Background

*LAMA2*-related muscular dystrophy is an autosomal recessive disorder caused by pathogenic variants in *LAMA2* gene (OMIM 156,225). *LAMA2* is located on 6q22.33 and encodes for laminin-α2 subunit of the heterotrimeric extracellular protein laminin-α2β1γ1 [1]. The clinical spectrum ranges from a severe, early-onset *LAMA2*-related congenital muscular dystrophy (*LAMA2*-CMD, OMIM 607,855) to a mild, childhood-onset autosomal recessive limb-girdle muscular dystrophy-23 (LGMDR23, OMIM 618,138) [2, 3]. The phenotypic difference seems to be related not only to the residual amount of laminin-α2, but also to the location and type of pathogenic variants [4, 5]. *LAMA2*-CMD is one of the most common congenital muscular dystrophies (CMDs) in the world, accounting for 36.4%-48% of CMD patients [6–8]. However, the prevalence of LGMDR23 is not fully known. Although there are a few retrospective, cross-sectional studies exploring the natural history and genetic variations of *LAMA2*-related muscular dystrophy [4–7], long-term and large-scale studies of the natural history and genotype–phenotype correlations are limited. As promising therapeutic approaches (such as upregulation of *LAMA1*, mini-agrin, and laminin-α1 LN-domain nidogen-1 (αLNNd)) are getting closer to clinical application [9–12], the definition of natural history endpoints along with clinically relevant outcome measures is essential for both clinical care planning and clinical trial readiness [13].

In this study, we designed a large national multicenter study of *LAMA2*-related muscular dystrophy patients in the Chinese population. Detailed clinical and genetic data were collected for analyzing the symptom onset, survival length, motor development and regression, occurrence of disease-related complications and genetic features. Our purpose was to identify valid and reliable information of the natural history and genotype–phenotype correlations of *LAMA2*-related muscular dystrophy.

## Results

**Fig. 1:**
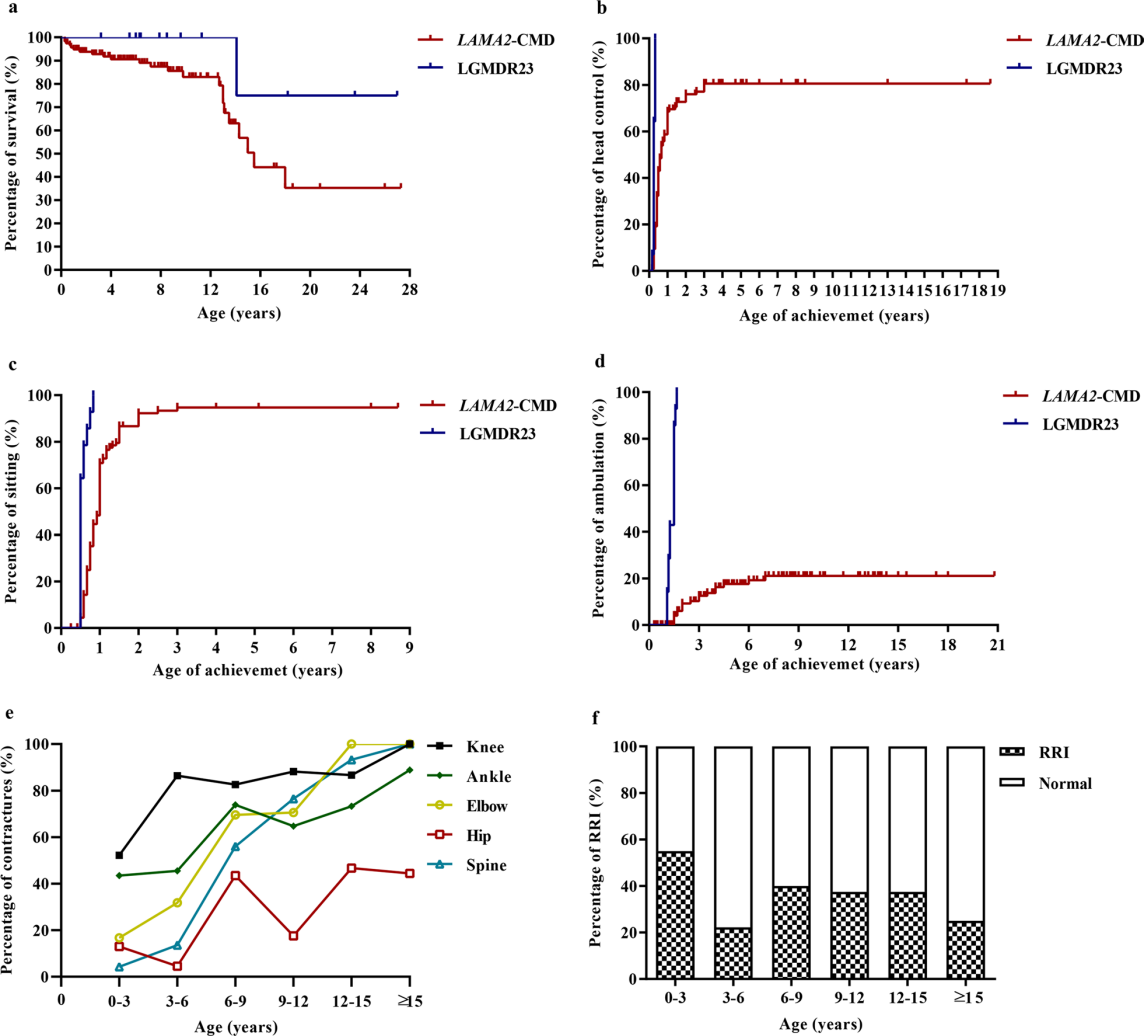
The clinical features in patients with *LAMA2*-related muscular dystrophy. **a** Kaplan–Meier survival analysis in patients with *LAMA2*-related muscular dystrophy. **b** Comparison of achievemet of head control in patients with *LAMA2*-related muscular dystrophy. **c** Comparison of achievemet of sitting in patients with *LAMA2*-related muscular dystrophy. **d** Comparison of achievemet of ambulation in patients with *LAMA2*-related muscular dystrophy. **e** Joint contractures in patients with *LAMA2*-CMD in different ages. **f** Recurrent respiratory infection in patients with *LAMA2*-CMD in different ages. *LAMA2-CMD LAMA2*-related congenital muscular dystrophy, *LGMDR23* autosomal recessive limb-girdle muscular dystrophy-23, *RRI* recurrent respiratory infection

The study cohort included 130 patients (116 *LAMA2*-CMD and 14 LGMDR23) (Additional files 1 and 2), 124 (95.4%) were Han Chinese, 79 were male, and 24 died (Fig. 1a). The median age at the last follow-up was 6.4 years (range 0.3–27.3 years) for *LAMA2*-CMD and 8.2 years (range 3.2–27.0 years) for LGMDR23 patients.

### Clinical characteristics

#### Onset of symptoms

The median age of symptom onset was 0.0 months (range 0.0–6.0 months) for *LAMA2*-CMD and 18.0 months (range 13.0–156.0 months) for LGMDR23 (Table 1). Early symptoms appeared within the first week of life in 75.9% (88/116) of *LAMA2*-CMD patients. The onset symptoms in *LAMA2*-CMD were variable combinations of severe muscle weakness (n = 114), hypotonia (n = 114), weak cry (n = 48), neonatal feeding difficulty (n = 30), neonatal respiratory difficulty (n = 9). Four patients were treated with mechanical ventilation in the neonatal period. In LGMDR23, the onset symptoms were myopathic gait (n = 10), difficulty running and jumping (n = 3) and epilepsy (n = 1).

**Table 1:** Clinical and demographic characteristics of *LAMA2*-related muscular dystrophy cohort

	*LAMA2*-CMD	LGMDR23
Age of onset, median (range), months	0.0 (0.0–6.0)	18.0 (13.0–156.0)
Age of last follow-up, median (range), years	6.4 (0.3–27.3)	8.2 (3.2–27.0)
Male, % (n)	62.1 (72/116)	50.0 (7/14)
Survivors, % (n)	80.2 (93/116)	92.9 (13/14)
Ambulation alone over 1.5 years old, % (n)	18.4 (18/98)	100 (14/14)
Regression of motor function, % (n)	31.2 (34/109)	7.1 (1/14)
Spinal deformity, % (n)	48.6 (54/111)	7.1 (1/14)
Central nervous system involvement		
Cognitive impairment, % (n)	10.3 (12/116)	0.0 (0/14)
Seizure, % (n)	9.5 (11/116)	35.7 (5/14)
Typical brain white matter alterations, % (n)	92.2 (95/103)	69.2 (9/13)
Respiratory involvement		
Neonatal respiratory difficulty, % (n)	7.7 (9/116)	0.0 (0/14)
Breathing difficulty during follow-up, % (n)	26.9 (29/108)	0.0 (0/14)
Feeding		
Neonatal feeding difficulty, % (n)	25.8 (30/116)	0 (0/14)
Chewing difficulty over 2 years old, % (n)	65.1 (53/90)	0.0 (0/14)
Swallowing difficulty over 2 years old, % (n)	65.1 (11/90)	0.0 (0/14)

*LAMA2-CMD LAMA2*-related congenital muscular dystrophy, *LGMDR23* limb-girdle muscular dystrophy-23

#### Motor development and regression

Kaplan–Meier survival analysis showed differences in the motor milestones of head control, independent sitting and ambulation between *LAMA2*-CMD and LGMDR23 (Fig. 1b–d). Among the 114 *LAMA2*-CMD older than 4 months, 87 (76.3%) achieved head control (65 achieving after 4 months old) at median age of 6.0 months (range 2.0–36.0 months), but 27 never achieved; delayed head control was observed in 80.7% ((65 + 27)/114) of *LAMA2*-CMD. Among the 107 *LAMA2*-CMD older than 10 months and one nine-month-old patient with independent sitting, 100 (92.6%) achieved independent sitting (51 achieving after 10 months old) at median age of 11.0 months (range 6.0–36.0 months), 8 never achieved; delayed independent sitting was observed in 54.6% ((51 + 8)/108) of *LAMA2*-CMD. Among the 98 *LAMA2*-CMD older than 18 months, 18 (18.4%) achieved independent ambulation (14 achieving after 18 months old) at median age of 27.0 months (range 18.0–84.0 months), 80 never achieved; delayed independent ambulation was observed in 95.9% ((14 + 80)/98) of *LAMA2*-CMD. In LGMDR23, all patients achieved independent ambulation at median age of 18.0 months (range 13.0–20.0 months). Five LGMDR23 had difficulty in running and jumping. Motor regression assessed in 109 *LAMA2*-CMD was observed in head control (n = 7), rolling (n = 9), independent sitting (n = 17) and ambulation (n = 9), at median (range) ages of 9.8 (6.8–11.0), 6.0 (3.8–12.0), 8.0 (4.1–19.0) and 8.0 (1.7–11.0) years, respectively. However, only one LGMDR23 gradually lost ambulation at 5.5 years of age.

#### Central nervous system involvement

Seizures occurred in 9.5% (11/116) of *LAMA2*-CMD patients (night with epilepsy and two with febrile seizures) and 35.7% (5/14) of LGMDR23 patients (three with epilepsy and two with febrile seizures). Only one LGMDR23 patient (P123) had a family history of epilepsy. The seizure types in *LAMA2*-CMD were broad, including focal seizures, generalized seizures, myoclonus, typical or atypical absence seizures. Three LGMDR23 patients showed focal seizures with paroxysmal blurry vision, headache and vomiting (n = 1), clonic seizure of the limb (n = 1) and facial twitching (n = 1). The results of electroencephalography showed slow wave in occipital and posterior temporal region. The median age of the first epileptic episode was 13 years (range 0.1–15.5 years) in *LAMA2*-CMD, and three LGMDR23 patients developed the first epileptic episode at 11, 14 and 23 years of age, respectively. In addition, two *LAMA2*-CMD patients died of status epilepticus. Delayed development of cognitive function (n = 10) and regression of cognitive function following epilepsy (n = 2) were found in *LAMA2*-CMD, but only four of them underwent Intelligence Quotient tests.

**Fig. 2:**
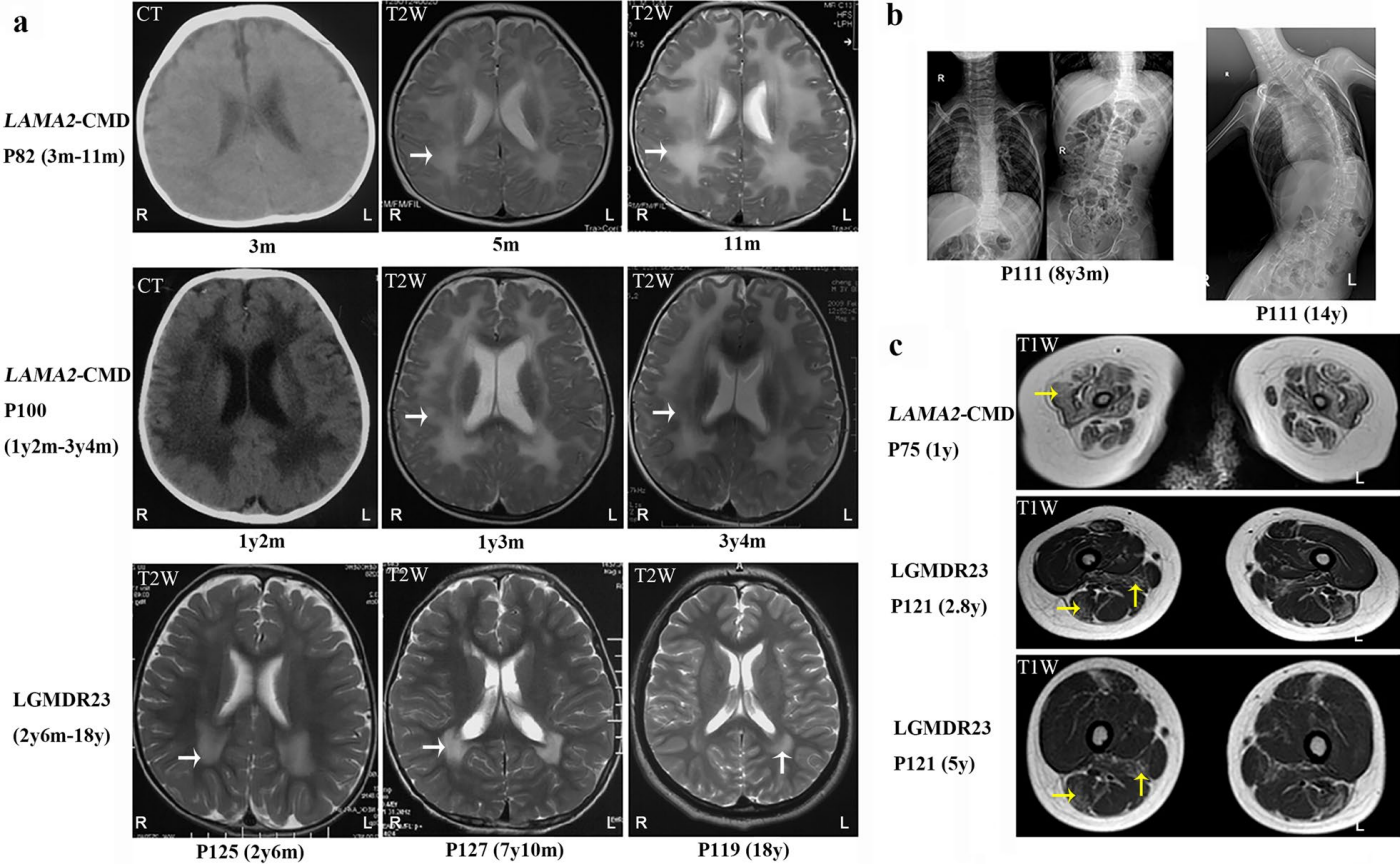
The image features in patients with *LAMA2*-related muscular dystrophy. **a** Changes of brain white matter (white arrows) in *LAMA2*-related muscular dystrophy in different ages. **b** Spinal deformity (scoliosis) of patient P111 with age. **c** Thigh muscle MRIs. Diffuse fatty infiltration (yellow arrows) in patient P75 (1y), fatty infiltration (yellow arrows) involved in the adductor magnus and long head of biceps femoris of patient P121 (2.8y and 5y). *LAMA2-CMD LAMA2*-related congenital muscular dystrophy, *LGMDR23* autosomal recessive limb-girdle muscular dystrophy-23

A total of 92.2% (95/103) of *LAMA2*-CMD patients showed widespread abnormal white matter hyperintensities on T2-magnetic resonance imaging (MRI) (Fig. 2a) as previously described [14]. In the other eight *LAMA2*-CMD, brain white matters on MRIs performed at ages of 5 days to 4 months were normal or focal changes, while without reexamination later. Except nine LGMDR23 patients with widespread abnormal white matter hyperintensities on T2-MRI, four LGMDR23 showed milder white matter changes predominately in the anterior and/or posterior horns of lateral ventricle (Fig. 2a). Brain MRIs of 53 *LAMA2*-related muscular dystrophy patients were reassessed, occipital pachygyria (18.7%, 10/53) and pontine hypoplasia (15.1%, 8/53) were found (Additional file 1). Among the ten patients with occipital pachygyria, only three had epilepsy, the correlation between occipital pachygyria and epilepsy was not observed.

#### Orthopedic complications

Joint contractures assessed in 109 *LAMA2*-CMD patients involved the knees at first, then the ankles, elbows and hips in sequence (Fig. 1e). They progressed rapidly during 6–9 years old with the rate of 82.6%, 73.9%, 69.6% and 43.5%, respectively. Scoliosis (Fig. 2b) occurred in 40.5% (45/111) of *LAMA2*-CMD at median age of 6.0 years (range 0.5–12.0 years), and lordosis occurred in 8.1% (9/111) of *LAMA2*-CMD at median age of 3.0 years (range 2.0–7.0 years). Pectus carinatum, pectus excavatum and hip dislocation were observed in 19.0% (22/116), 12.1% (14/116) and 9.5% (11/116) of *LAMA2*-CMD, respectively. However, only four LGMDR23 had either one or two symptoms of tightened Achilles tendons, contractures of ankles and knees, and mild scoliosis.

#### Respiratory involvement

In total, 58.9% (63/107) of *LAMA2*-CMD and 35.7% (5/14) of LGMDR23 patients had a history of recurrent respiratory infection, mainly at ages of 0–3 years and 6–15 years (Fig. 1f). Eighteen *LAMA2*-CMD patients died of respiratory failure following severe pneumonia. Besides respiratory difficulty in the neonates and following severe pneumonia, 11 *LAMA2*-CMD developed dyspnea at median age of 11 years (range 6–24 years). Pulmonary function tests and/or polysomnographic examination were performed in 12 *LAMA2*-CMD at 4–18 years old, the main findings were respiratory insufficiency with restrictive ventilation dysfunction and nocturnal hypoxemia. However, only four *LAMA2*-CMD underwent mechanical ventilation during follow-up (at ages of 8.2, 11.2, 12.6 and 18.9 years). Although no LGMDR23 had symptoms of breathing difficulty, the pulmonary function test of P126 showed moderate to severe restrictive lung disease at 8.7 years old.

#### Cardiac involvement and feeding problem

Electrocardiography (ECG) and echocardiogram were performed in 63 patients (Additional file 1). ECG showed normal ECG or nonspecific changes. Echocardiogram mainly showed normal function or mild valvular regurgitation, except one decreased left ventricular ejection fraction (LVEF) of 45% in P80 at 1.8 years old. The LVEF became normal after treatment with captopril (an angiotensin converting enzyme inhibitor) for two years. In *LAMA2*-CMD patients over two years old, feeding problems such as chewing difficulty (58.9%, 53/90) and swallowing difficulty (12.2%, 11/90) were common. Only patients P87 and P94 underwent nasogastric feeding. P102 and P112 died of malnutrition and cachexia following swallowing difficulty at 13.5 and 18 years old, respectively.

#### Survival

Twenty-four patients (23 *LAMA2*-CMD and one LGMDR23) (Additional file 2) died at median age of 7.9 years (range 0.3–18.0 years). The remaining 106 patients were still alive at the last follow-up with median age of 6.5 years (range 0.5–27.3 years). The accumulative survival rate of *LAMA2*-CMD patients was approximately 50% by the age of 15 years (Fig. 1a). There were two peaks in mortality: the first year of life (13/24) and 12–15 years old (8/24). The causes of death in *LAMA2*-CMD included respiratory failure following severe pneumonia (18/23, 78.3%), status epilepticus (2/23, 8.7%) and malnutrition following swallowing difficulty (2/23, 8.7%). One *LAMA2*-CMD (P84) and one LGMDR23 (P127) died of sudden death with unknown reason, but the *LAMA2*-CMD patient had sustained vomiting one week before death.

#### Other diagnostic tests

Immunohistochemistry staining of laminin-α2 showed complete laminin-α2 deficiency in 19 *LAMA2*-CMD, and partial deficiency in three *LAMA2*-CMD and two LGMDR23 patients. Therefore, complete laminin-α2 deficiency was associated with *LAMA2*-CMD (*p* = 0.036). A total of 3/19 patients with complete laminin-α2 deficiency and 3/5 patients with partial laminin-α2 deficiency achieved ambulation (*p* = 0.078). The thigh muscle MRIs of 14 *LAMA2*-CMD showed diffuse fatty infiltration in the adductor magnus, gluteus maximus, quadriceps femoris muscle and biceps femoris (Fig. 2c). For four LGMDR23, fatty infiltration in the mid-thigh level frequently and selectivity involved in the adductor magnus and long head of biceps femoris (Fig. 2c).

### Clinical characteristics correlations

Two peaks in mortality, the first year of life and 12–15 years old, were consistent with peaks of recurrent respiratory infection. In *LAMA2*-CMD, survival was related to head control (*p* = 0.022) and sitting ability (*p* = 0.010), and epilepsy was associated with lower rate of survival (*p* = 0.015) (Additional file 3). In *LAMA2*-CMD, spinal deformity was associated with motor regression (*p* < 0.001). Compared with non-ambulatory *LAMA2*-related muscular dystrophy patients, epilepsy was associated with the ambulatory group (*p* = 0.008). Seizures were more frequent in LGMDR23 (35.7%) than in *LAMA2*-CMD (9.5%).

### Genetic characteristics

**Fig. 3:**
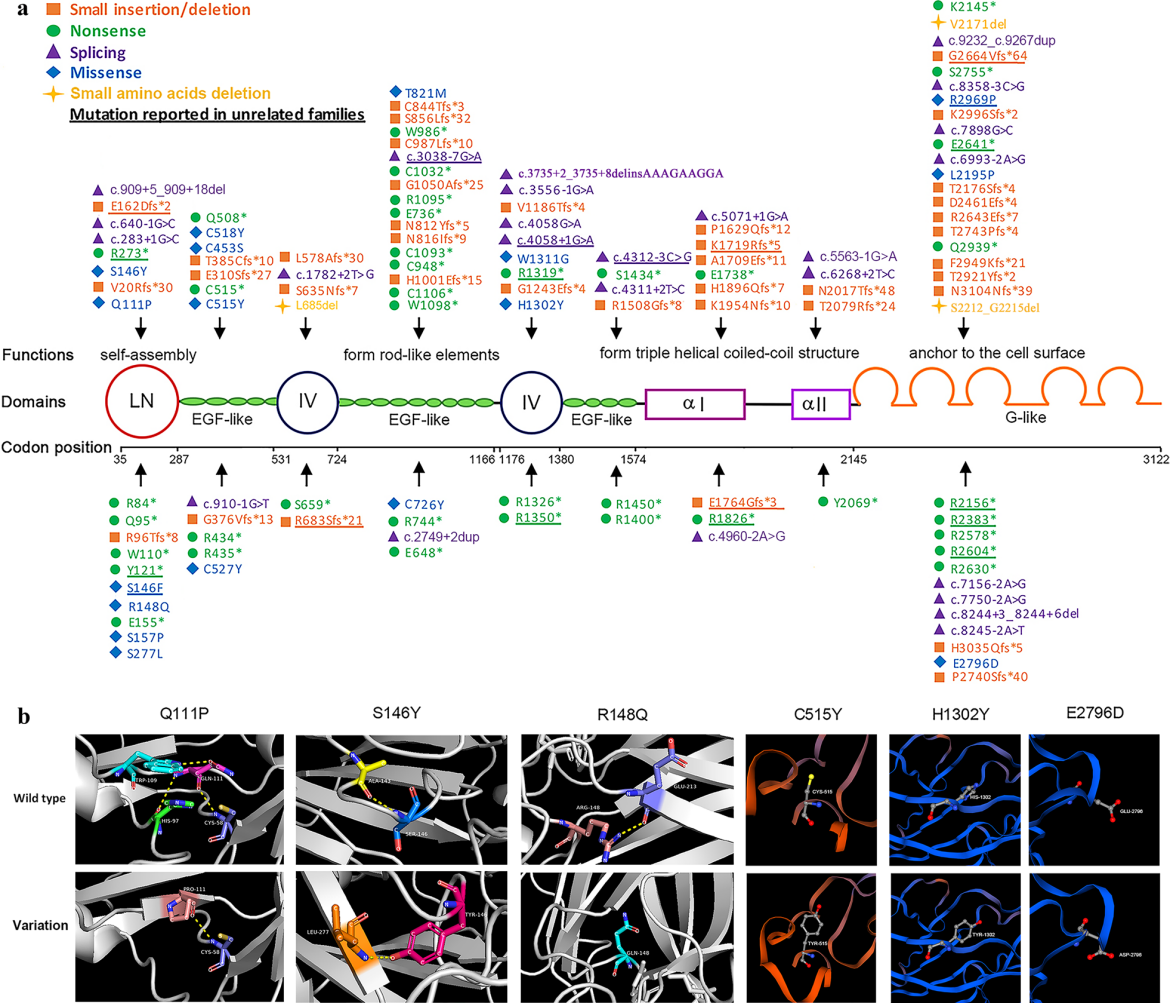
Schematic of the *LAMA2* disease-causing variants. **a** The domain structure of laminin-α2 is illustrated. Disease-causing variants (except copy number variations) including 44 novel disease-causing variants and 36 disease-causing variants first reported in our previous works in our cohort are above the chain, and other known disease-causing variants are below. **b** Four novel missense variants (p.Q111P, p.S146Y, p.C515Y, p.H1302Y) and two known missense variants (p.R148Q, p.E2796D) are predicted by modeling three-dimension structure of the protein. *α I* laminin helical coiled-coil domain I, *α II* laminin helical coiled-coil domain II, *IV* laminin IV type A1 or A2, *LN* laminin N-terminal

**Fig. 4:**
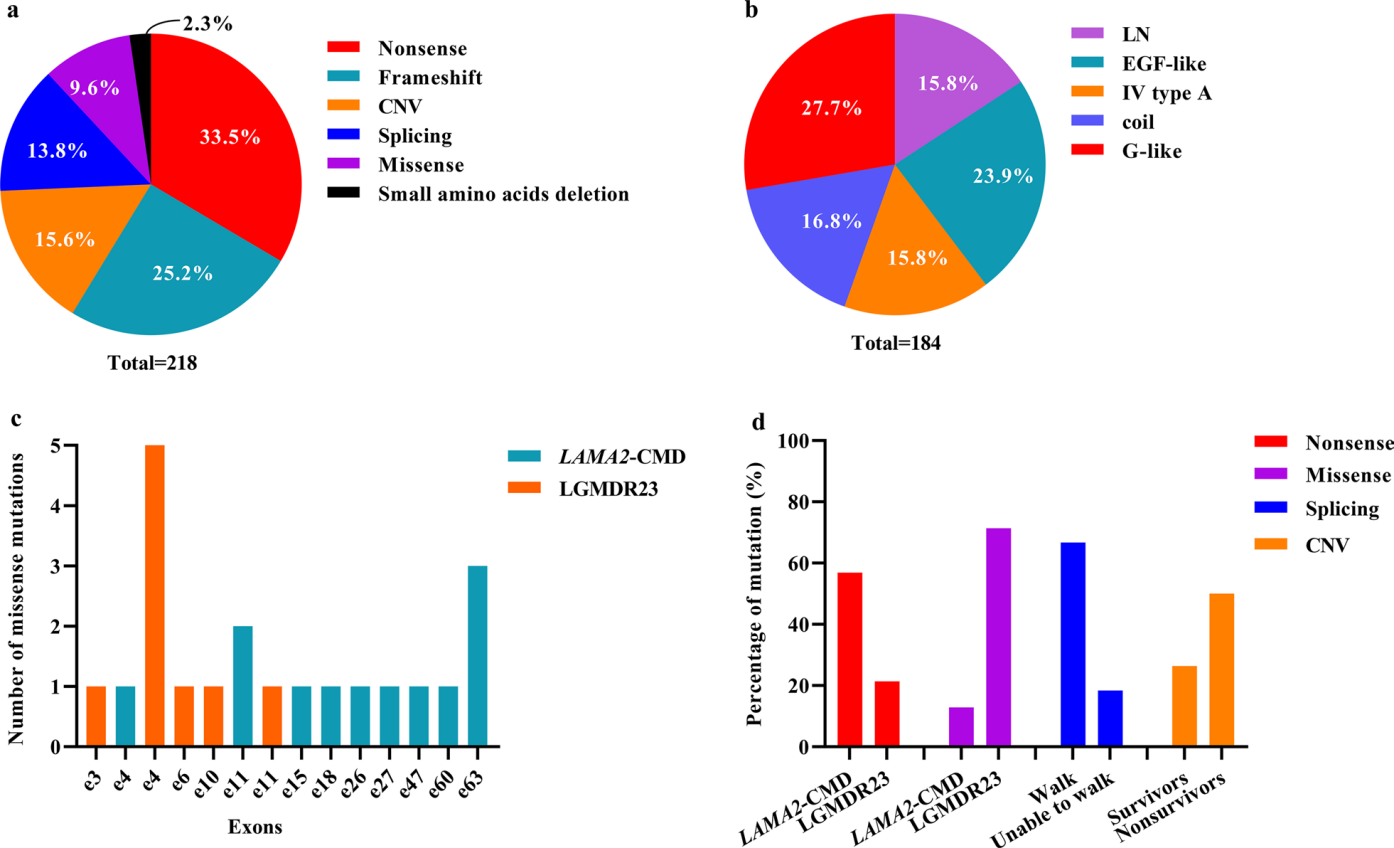
Genetic characteristics and genotype–phenotype correlations in *LAMA2*-related muscular dystrophy. **a** Six types of disease-causing variants in 218 alleles. **b** Distribution of 184 allele mutations (except CNVs). **c** Distribution of missense disease-causing variants in LGMDR23 and *LAMA2*-CMD. **d** Nonsense in *LAMA2*-CMD patients, missense disease-causing variants in LGMDR23 patients, splicing disease-causing variants in ambulatory *LAMA2*-CMD patients and CNVs in nonsurvivors were more frequent. *CNVs* copy number variations; *Coil* laminin helical coiled-coil domain, *IV type A* laminin IV type A1 or A2, *LN* laminin N-terminal, *LAMA2-CMD LAMA2*-related congenital muscular dystrophy, *LGMDR23* autosomal recessive limb-girdle muscular dystrophy-23, *Splicing* splicing disease-causing variant

Pathogenic variants were detected in 130 patients from 111 families. Five probands’ parents were consanguineous; proband P78 and patient P19 were second cousins sharing the same great grandparent. We identified 46 novel and 97 known pathogenic variants in *LAMA2* gene (Fig. 3a and Additional file 4). Among the known pathogenic variants, 54 were first reported in our previous works [8, 14–16]. The disease-causing variants in 218 (n = 111 × 2 + 1 – 5) alleles included nonsense (73/218, 33.5%), frameshift (55/218, 25.2%), CNVs (34/218, 15.6%), splicing (30/218, 13.8%), missense (21/218, 9.6%), and small amino acid deletions (5/218, 2.3%) (Fig. 4a).

It should be pointed out that based on the American College of Medical Genetics and Genomics (ACMG) variant-classification, among the four novel and 13 known missense variants, 15 were classified as variant of uncertain significance (VUS), one as likely pathogenic variant, and one as pathogenic variant (Additional file 5). Three novel variants p.Q111P, p.S146Y and p.C515Y were predicted to be damaging by four softwares, p.H1302Y was predicted to be damaging by three softwares (Additional file 5). These amino acid substitutions were on the conserved amino acid sequences, and were predicted to be damaging by modeling three-dimension structure of the protein (Fig. 3b). Among the 13 known missense variants, 12 were predicted to be damaging, but p.E2796D was predicted to be tolerated (Additional file 5). However, p.E2796D was located on the hotspot pathogenic variants region of G-like domain, the glutamic acid residue was conserved, the variant was present in population with 0.00016 (Exome Aggregation Consortium) and in accord with segregation in the family, the patient was clinically diagnosed with *LAMA2*-CMD without CNV. Therefore, p.E2796D was identified as a pathogenic variant. Notably, the variant c.2462C > T with a conflicting pathogenicity was classified as ACMG: 3 (VUS) in the supplementary data, while was still considered to be a disease-causing variant in this study. In our cohort, it was homozygous in two siblings with *LAMA2*-CMD from consanguineous parents, and was co-segregating in the family. c.2462C > T was predicted to be damaging by four tolerance predictors, was estimated with a low frequency ranging from 0.00080 (1000 Genomes Project) to 0.00238 (NHLBI Exome Sequencing Project (ESP) Exome Variant Server). Moreover, there were another three unrelated patients with *LAMA2*-related muscular dystrophy carrying the variant (https://www.ncbi.nlm.nih.gov/clinvar/variation/162584/). Furthermore, a missense variant at the same residue (T821P) has been reported as pathogenic/likely pathogenic without conflicts [2]. Two variants, c.4058G > A changing the last base of exon 27 and c.7898G > C changing the last base of exon 56, were predicted to produce abnormal splice sites and were classified as splicing variants.

Most of the 184 mutant alleles (except CNVs) were distributed in the laminin N-terminal (LN) (29/184, 15.8%), EGF-like domain (44/184, 23.9%), and G-like domain (51/184, 27.7%) (Fig. 4b). The 34 CNVs were mainly distributed in the LN and G-like domains. Six high-frequency pathogenic variants in Han Chinese patients were c.7147C > T (p.R2383*) (n = 11), exon 4 deletion (n = 10), c.5156_5159del (p.K1719Rfs*5) (n = 8), c.2049_2050del (p.R683Sfs*21) (n = 5), c.7921G > T (p.E2641*) (n = 5) and c.4048C > T (p.R1350*) (n = 5). Deletion of exon 4 was a founder pathogenic variant in Han Chinese patients [15].

### Genotype–phenotype correlations

Genotype–phenotype correlations in patients with *LAMA2*-related muscular dystrophy were shown in Additional file 6 and Fig. 4c, d. Most of splicing variants (96.7%, 29/30) were found in the *LAMA2*-CMD patients but only one in the LGMDR23 patients. In 98 patients with *LAMA2*-CMD over 18 months old, splicing variants were found in 66.7% (12/18) of ambulatory patients and 17.5% (14/80) of non-ambulatory patients. Additionly, in 107 patients with *LAMA2*-CMD over 10 months old along with one nine-month-old patient with sitting, splicing variants were found in 12.5% (1/8) of patients never achieving sitting, 15.6% (8/51) of patients achieving sitting over than 10 months old, and 38.7% (19/49) of patients achieving sitting within 10 months old. Therefore, splicing variants tended to be associated with a relatively mild phenotype in *LAMA2*-CMD. Nonsense was more frequent in *LAMA2*-CMD patients (56.9%, 66/116) than in LGMDR23 patients (21.4%, 3/14), consequently, combinations containing nonsense (frameshift + nonsense, nonsense + nonsense, nonsense + CNV, and nonsense + splicing) were mainly found in *LAMA2*-CMD patients. The most high-frequency nonsense c.7147C > T was only detected in *LAMA2*-CMD patients. However, missense variants were more frequent in LGMDR23 patients (71.4%, 10/14) than in *LAMA2*-CMD patients (12.9%, 15/116), especially missense pathogenic variants in exon 4 (Fig. 4c, d). The proportion of CNV as well as frameshift was similar between *LAMA2*-CMD and LGMDR23. However, 26.4% of survivors and 50.0% of nonsurvivors had CNV, showing that CNV was associated with lower rate of survival (*p* = 0.029). On the whole, the damages of the types of disease-causing variants in *LAMA2*-MD were in the order: nonsense/frameshift/CNV > splicing > missense variants.

## Discussion

Though muscular dystrophies due to *LAMA2*-related muscular dystrophy is relatively rare, they cover a distinct clinical presentations and severity with high disability and disease burden, and the further mechanism how the pathogenic variants affect the function of laminin-α2 leading to clinical heterogeneity is still unclear. Recently, two natural history studies on 46 *LAMA2*-related muscular dystrophy pediatric patients in the Dubowitz Neuromuscular Centre [5] and 24 *LAMA2*-related muscular dystrophy patients in National Institutes of Health [17] provided useful information towards trial readiness. However, international multicenter studies with detailed knowledge on long-term progression of disease as well as genotype–phenotype correlations remain challenging. Here, we report a comprehensive natural history and genetic analysis in the largest *LAMA2*-related muscular dystrophy cohort thus far, to address clinical and genotypic predictors of phenotypes and disease progression. This cohort represents a subgroup of particular medical needs due to severe clinical manifestations.

In the current study, we have reported that the symptom onset of LGMDR23 patients at 1–3 years old was common. This contrasted with previous studies showing the ages of symptom onset ranging from 1–3 years old to 17.0 ± 7.5 years old in LGMDR23 patients [18–20]. This might be due to the later recognization of the mild clinical presentations, limited number and different demography of the recruited patients. Therefore, for patients with symptoms of muscular dystrophy in childhood, the possibility of LGMDR23 should be considered and brain MRI scan should be further performed. Similar with the variable phenotypes in *LMNA*-related muscular dystrophy and POMT1-related disorders [21, 22], *LAMA2*-CMD and LGMDR23 could be viewed as a continuum spectrum. *LAMA2*-CMD patients presented with more earlier and severe muscle weakness and hypotonia at onset. Therefore, based on the age and symptoms of onset, it was relatively easy to distinguish the two groups. However, the detailed data between them were little known, especially the disease progression.

The stage of motor function directly affected patients’ quality of life. In *LAMA2*-CMD, head control and independent sitting were achieved in 76.3% and 92.6%, while delayed in 80.7% and 54.6%, respectively; only 18.4% of *LAMA2*-CMD achieved ambulation. Notably, motor regressions were frequent in *LAMA2*-CMD [23], and occurred concurrently with joint contractures and spinal deformity during the critical period of 6–9 years old. These suggested that most *LAMA2*-CMD patients could not cope with everyday activities, and needed help and care. Preventing or delaying orthopedic complications with physical therapy in the early stage could be beneficial for improving motor function [24, 25]. Orthopedic surgery might be needed for severe contractures and spinal deformities [26, 27]. More over, achievements of head control and independent sitting were predictors of longer survival in *LAMA2*-CMD. However, LGMDR23 showed mild and slowly progressive muscle weakness. Therefore, the evaluation of motor function is necessary for the better understanding of the two phenotypes and the better management of disease.

We only found complete laminin-α2 deficiency was associated with *LAMA2*-CMD, but did not observe the reported histochemical difference between ambulatory and non-ambulatory patients [4, 6, 28], this might due to the limited number of muscle biopsies available. Considering gene test becoming more and more precise, immunohistochemical staining not always corresponding to the disease severity and the limited availability of muscle biopsy on infantile patients, muscle biopsy may not be necessary for the diagnostic criteria. The pattern of muscle involvement on thigh MRI showed that fatty infiltration in the mid-thigh level predominantly and severely involved the anterior and posterior thigh muscles in *LAMA2*-CMD, while selectively involved the adductor magnus and long head of biceps femoris in LGMDR23. Thigh muscle MRI might be useful to predict motor function and assess disease progression, and was worthy of further investigations [29, 30].

Other disease-related complications, including respiratory involvement, seizures and feeding difficulty, not only affected the quality of life, but also affected the survival. Although early death was reported in *LAMA2*-CMD [4, 31, 32], long term survival has not been well documented in large cohorts. A total of 19.8% of *LAMA2*-CMD patients died during follow-up, the accumulative survival rate by the age of 15 years was approximately 50%. The causes of death in *LAMA2*-CMD were respiratory failure and pneumonia (78.3%), status epilepticus (8.7%) and malnutrition (8.7%). Respiratory insufficiency could manifest with recurrent chest infection starting from first two years of life [5], and the need for ventilation longer than 4 weeks was reported as a predictor of sudden death [33]. Therefore, *LAMA2*-CMD patients should receive standardized respiratory care according to current guidelines [34]. Seizures were reported in 8–50% of *LAMA2*-CMD [35–37], while were found in 35.7% of LGMDR23 and 9.5% of *LAMA2*-CMD patients in this study. Focal seizures with visual aura and autonomic signs including nausea might not be identified, and electroencephalography was not easily accessed to for the older patients, the incidence of seizures in *LAMA2*-CMD might be undervalued. It was worth mentioning that 5/9 *LAMA2*-CMD patients with epilepsy and all three LGMDR23 patients with epilepsy showed their first epileptic episode after age of 10 years, indicating that epilepsy might be more frequent in older children with *LAMA2*-related muscular dystrophy. Contrary to previous reports [38, 39], no correlation between occipital pachygyria and epilepsy was found in our cohort. Even though, epilepsy should be monitored, especially in older children with *LAMA2*-related muscular dystrophy. Malnutrition caused by feeding difficulty was also a common problem in *LAMA2*-CMD. In previous studies, 47.4% of *LAMA2*-CMD patients had feeding difficulty and required gastrostomy or nasogastric feeding before five years old [4, 40]. However, few *LAMA2*-CMD patients in this study underwent nasogastric feeding. Therefore, weight monitoring from early infancy and swallowing evaluation should focus on addressing feeding difficulties to minimize the failure to thrive. In a word, early prevention and interventions of complications were essential for the better quality of life and longer survival of *LAMA2*-CMD patients.

Splicing variant was previously thought to be more common in patients with LGMDR23 [2]. However, we found splicing variant occurred mainly in patients with *LAMA2*-CMD in the current study. Moreover, splicing variant tended to be associated with a relatively mild phenotype in *LAMA2*-CMD. Leaky splicing was reported to reduce the symptoms and self-improve clinical phenotype in some genetic diseases such as spinal muscular atrophy and neurofibromatosis type 1 [41, 42]. The impact of the different splicing variants might be related with a leaky splicing or other mechanisms which should be further studied. Recently, the adeno-associated virus carrying clustered regularly interspaced short palindromic repeats (CRISPR)-Cas9 was reported to correct a splice-site pathogenic ariant in dy^2J^/dy^2J^ mouse [43]. Patients with splicing variants might benefit from the splice modulating therapy in the future. We also found that *LAMA2*-CMD was related to nonsense variants, *LAMA2*-related muscular dystrophy with nonsense variants such as c.7147C > T (p.R2383*) might benefit from the stop codon readthrough. We also confirmed that missense pathogenic variants was associated with LGMDR23, so missense pathogenic variants could be a predictor of the milder phenotype. Nearly ten percent of the pathogenic variants involved exon 4, and missense and nonsense pathogenic variants in exon 63 were only found in patients with *LAMA2*-CMD. Based on these findings, we speculate that exon 4 is important for laminin-211 self-assembly and exon 63 plays an important role in the function of the G-like domain. Potential therapeutic interventions including splice modulation, upregulation of the *LAMA1* gene, mini-agrin and αLNNd are in development for *LAMA2*-related muscular dystrophy [9, 10, 38]. More knowledge of the genotype–phenotype correlations and the pathomechanism will lead to more discoveries for the therapy.

*Limitations:* This study has provided useful data regarding natural history and genetic features of *LAMA2*-related muscular dystrophy with some limitations. Even though the number of patients was substantial, most of patients included in the current study were pediatric patients with *LAMA2*-MD, and only limited data from adolescent and adult patients were available, which might affect the assessments especially for complications. Moreover, the number of patients with the LGMDR23 was relatively small. Considering a relative disproportion (116/14) between *LAMA2*-CMD and LGMDR23 subtypes, and the necessity of more LGMDR23 patients for adequate statistical power, we just did descriptive statistical analysis between the two subgroups. Finally, the number of muscle biopsies available was limited, the study of correlation between histochemical difference of laminin-α2 with the disease severity was affected.

## Conclusion

In summary, in the current study, the natural history and genetic features of *LAMA2*-related muscular dystrophy were characterized. The study provides important information and basis for differentiating *LAMA2*-related muscular dystrophy subtypes and different focus of multidisciplinary management of *LAMA2*-related muscular dystrophy subtypes. These results should be valuable to future therapeutic trials and be crucial for future discovery of gene therapies.

## Methods

### Patients and study design

*LAMA2*-related muscular dystrophy patients were enrolled between January 2003 and March 2021 at ten research centers in China. Clinical evaluations at enrollment and annual follow-up visits were collected, the medical records were retrospectively reviewed. The inclusion criteria were a clinical and genetical diagnosis of *LAMA2*-related muscular dystrophy characterized by muscle weakness or hypotonia, delayed motor developmental milestones, hypercreatine kinasemia, abnormal white matter hyperintensities on T2-MRI, and pathogenic variants in *LAMA2* gene. Based on the age of onset and disease severity, patients were divided into two subgroups: *LAMA2*-CMD and LGMDR23 [3]. Patients belonged to *LAMA2*-CMD if they presented with hypotonia and muscle weakness within the first six months of life, and/or delayed motor milestones in the first year of life. Patients who had mild proximal muscle weakness but with normal motor milestones in the first year of life and independent ambulation were classified as LGMDR23. Achieving or never achieving head control after 4 months old, independent sitting after 10 months old, or independent walking after 18 months old were considered as delayed motor milestones.

### Clinical data and outcome measures

The primary clinical outcome measures included the onset, motor development and regression, multiple system complications and survival length. Routine laboratory data including serum creatine kinase, ECG, echocardiogram, brain MRI, thigh muscle MRI, and electromyography (EMG) were collected. Muscle biopsies were also performed, and laminin-α2 expression was detected with mAb 1922 (1:5000,100 μL; 5H2, Merck Millipore, Darmstadt, Germany). The correlations between phenotypes of the disease and clinical outcomes were analyzed.

### LAMA2 pathogenic variants analysis

Genomic DNA was extracted from peripheral blood lymphocytes. Direct sequencing of *LAMA2* gene before year 2013 and a custom-designed modified next-generation sequencing (NGS) starting in year 2013 were performed to identify the pathogenic variants of *LAMA2* gene [14]. Multiplex ligation-dependent probe amplification (MLPA) (SALSA MLPA kit P391-A1/P392-A1, MRC-Holland, Amsterdam, The Netherlands) and custom-designed high-resolution *LAMA2*-targeted array-based comparative genomic hybridization (SurePrint G3 Microarray, 4 × 180 K) were performed to identify CNV [15]. The pathogenicity of identified variants was evaluated based on the ACMG guidelines. The pathogenicity of missense variants was scored using Polyphen-2, SIFT, Mutation Taster and CADD based on the recommendation of the ACMG guidelines. Moreover, the genotype characteristics and genotype–phenotype correlations (the secondary outcomes) were analyzed.

### Statistical analysis

The non-normal distributed data were expressed as median (range). Pearson χ^2^ test, Fisher’s exact test, and Mann–Whitney *U* test were used wherever appropriate. GraphPad Prism 5 software (GraphPad Software, La Jolla, CA) were used to create Kaplan–Meier survival curves and graphs of clinical and genetic features. Statistical analyses were performed using SPSS (version 19.0; IBM-SPSS, Chicago, IL). Two-sided *p* < 0.05 was considered to be statistically significant.

## Supplementary Information

**Additional file 1.** Clinical findings of patients with *LAMA2*-related muscular dystrophy.

**Additional file 2.** The ages of patients with *LAMA2*-related muscular dystrophy.

**Additional file 3.** Effect of motor and epilepsy on survival of *LAMA2*-CMD.

**Additional file 4.** Genetical analysis of patients with *LAMA2*-related muscular dystrophy.

**Additional file 5.** Pathogenicity analysis of 17 missense variants.

**Additional file 6.** Genotype-phenotype correlations in patients with *LAMA2*-related muscular dystrophy.

## Data Availability

The datasets used during the current study are available from the corresponding author on reasonable request. All data relevant to the study are included in the article and its supplementary information files.

## Abbreviations

αLNNd: Laminin-α1 LN-domain nidogen-1
ACMG: American college of medical genetics and genomics
CMD: Congenital muscular dystrophy
CNVs: Copy number variations
CRISPR: Clustered regularly interspaced short palindromic repeats
ECG: Electrocardiography
EMG: Electromyogram
LN: Laminin N-terminal
*LAMA2*-CMD: *LAMA2*-related congenital muscular dystrophy
LGMDR23: Autosomal recessive limb-girdle muscular dystrophy-23
LVEF: Left ventricular ejection fraction
MLPA: Multiplex ligation-dependent probe amplification
MRI: Magnetic resonance imaging
NGS: Next-generation sequencing
VUS: Variant of uncertain significance
