# Supplementary material for: Natural history and genetic study of LAMA2-related muscular dystrophy in a large Chinese cohort
Source: Orphanet J Rare Dis. 2021 Jul 19;16:319. doi: 10.1186/s13023-021-01950-x (PMC8287797; doi:10.1186/s13023-021-01950-x)
Supplement: Supplementary file 1 — Additional file 1. Clinical findings of patients with LAMA2-related muscular dystrophy. [file 13023_2021_1950_MOESM1_ESM.docx]

**Additional file 1.** Clinical findings of patients with *LAMA2*-related muscular dystrophy

| **Patient/ sex/age** | **Age at onset** | **Symptoms of onset** | **Motor achievement (age)** | **Regression of motor (age)** | **Contracture** | **Spinal deformity (age)** | **Respiratory involvement** | **ECG changes** | **UCG changes** | **Feeding** | **Regular rehabilitation** | **Intellect/ seizure** | **Highest serum CK U/L (age)** | **EMG myopathic changes (age)** | **IH staining of**  **laminin α2 (age)** | **Brain T2- weighted MRI (age)** | **Thigh muscle MRI** | **Others** |
| --- | --- | --- | --- | --- | --- | --- | --- | --- | --- | --- | --- | --- | --- | --- | --- | --- | --- | --- |
| P1/M/3 m | Birth | Muscle weakness, hypotonia | Can’t raise head | Na | Na | Na | RRI, severe pneumonia | Na | Na | Na | Na | Normal intellect/ no seizure | Na | Na | Na | Na | Na | Died at 3 m following severe pneumonia |
| P2/F/3 m | Birth | Muscle weakness, hypotonia | Can’t raise head | Na | Na | Na | RRI, severe pneumonia | Na | Na | Na | Na | Normal intellect/ no seizure | Na | Na | Na | Na | Na | Case 1’s elder sister, died at 3 m following severe pneumonia |
| P3/M/5 m | Birth | Muscle weakness, hypotonia, weak cry | Can’t raise head | No | Ankle | No | RRI, severe pneumonia | Na | Normal | Na | Na | Normal intellect/ no seizure | 4110 (2 days) | + (1 m) | Na | Focal changes (10 d) | Na | Case 1’s elder brother, died at 5 m following severe pneumonia, pectus excavatum |
| P4/M/6 m | Birth | Muscle weakness, hypotonia, weak cry | Can’t raise head | No | Knee | No | No | Normal | Normal | No |  | Normal intellect/ no seizure | 3761 (18 d) | +, MNCMAPA reduced (1 m) | Na | WMH | Na |  |
| P5/F/8 m | 1 m | Muscle weakness, hypotonia, weak cry | Can’t raise head | No | Ankle | No | RRI, severe pneumonia | Na | Na | Na | Na | Normal intellect/ no seizure | Na | Na | Na | Na | Na | Tajikistan, died at 8 m following severe pneumonia |
| P6/M/8 m | Birth | Muscle weakness, hypotonia | Can’t raise head | No | Ankle | No | No | Sinus tachycardia | Na | No | No | Normal intellect/ no seizure | 3159 (5 m) | Na | Na | WMH | Na |  |
| P7/F/8 m | Birth | Muscle weakness, hypotonia, weak cry, FD | HC (6 m) | No | Knee, ankle | No | No | Normal | Atrial septal defect (1 m) | FD at birth | Yes | Normal intellect/ no seizure | 26570 (3 days) | Na | Na | Normal (5 d) | Na |  |
| P8/M/9 m | Birth | Muscle weakness, hypotonia, weak cry, FD | S (9 m) | Na | No | No | Na | Na | Na | FD at birth | Na | Normal intellect/ no seizure | 5354 (3 m) | + | Na | WMH (4 m) | Na |  |
| P9/F/9 m | 3 m | Muscle weakness, hypotonia | Can’t raise head | Na | Na | Na | RRI, severe pneumonia | Na | Na | Na | Na | Normal intellect/ no seizure | Na | Na | Na | Na | Na | Died at 9 m following severe pneumonia |
| P10/M/1.0 y | Birth | Muscle weakness, hypotonia | S (11 m) | Na | Knee | No | RRI, severe pneumonia | Na | Na | Na | Na | Normal intellect/ no seizure | Na | Na | Na | WMH | Na | Arab, consanguineous parents, died at 1 y following severe pneumonia |
| P11/F/1.0 y | Birth | Muscle weakness, hypotonia | HC (7 m), S (8 m) | No | No | No | No | Sinus tachycardia | Normal | No | Yes | Normal intellect/ no seizure | 2155 (7m) | + (7 m) | Na | WMH (7 m) | Na |  |
| P12/M/1.2 y | Birth | Muscle weakness, hypotonia | HC (5 m), S (8 m) | No | Knee, ankle | No | RRI | Normal | PFO (4 m) | No | Yes | Intellectual delay/ no seizure | 3600 (4 m) | + (4 m) | Na | Focal changes (4 m) | Na |  |
| P13/M/1.3 y | Birth | Muscle weakness, hypotonia, weak cry | HC (9 m), S (1 y) | No | Knee, hip | No | Na | Na | Na | No | Na | Intellectual delay/ no seizure | 1807 (1.5 y) | +, MNCV reduced | Na | WMH | Na |  |
| P14/F/1.3 y | 1 m | Muscle weakness, hypotonia, weak cry, FD | Can’t raise head | No | Ankle | No | Na | Na | Na | FD from 20 days | Na | Normal intellect/ no seizure | Na | Na | Na | Na | Na | Tajikistan, Case 5’s elder sister, pectus excavatum |
| P15/M/1.3 y | 5m | Muscle weakness, hypotonia | HC (4 m) | No | No | No | No | Normal | Patent ductus arteriosus (1 m) | No | No | Normal intellect/ no seizure | 57985(birth) | Na | Na | Na | Na |  |
| P16/F/1.4 y | Birth | Muscle weakness, hypotonia | HC (1.4 y), S (7 m) | No | Knee | No | No | Na | Na | No | No | Normal intellect/ no seizure | 5536 (10 m) | MNCV reduced (10 m) | Na | WMH (10 m) | Na |  |
| P17/F/1.5 y | Birth | Muscle weakness, hypotonia, FD | HC (8 m), S (1.3 y) | No | No | No | RRI | Normal | Normal | FD at birth | Yes | Intellectual delay/ no seizure | 87452 (birth) | Na | Na | WMH (1.5 y) | Na |  |
| P18/F/1.5 y | Birth | Muscle weakness, hypotonia, weak cry | HC (5 m), S (1 y) | Na | Ankle | No | RRI, severe pneumonia | Na | Na | No | Na | Normal intellect/ no seizure | Na | Na | Na | WMH | Na | Died at 1.5 y following severe pneumonia |
| P19/F/1.6 y | 3 m | Muscle weakness, hypotonia | Can’t raise head | No | Ankle | No | No | Na | Na | No | No | Normal intellect/ no seizure | 6567 (6 m) | + | Na | WMH (6 m) | Na |  |
| P20/M/1.7 y | Birth | Muscle weakness, hypotonia, weak cry | HC (3 m), S (1 y) | No | Knee, hip | No | Na | Right bundle branch block | Normal | Constipation | Na | Intellectual delay/ no seizure | 2493 (1.7 y) | + | Na | WMH (7 m) | Na |  |
| P21/F/1.8 y | Birth | Muscle weakness, hypotonia | HC (6 m), S (1.5 y) | No | Knee | No | No | Normal | Atrial septal defect (8 m) | No | Yes | Normal intellect/ no seizure | 26909 (birth) | Na | Na | Focal changes (9 d) | Na | Hypoglycaemia at birth, pectus excavatum, dislocation of hip |
| P22/F/1.9 y | 6 m | Muscle weakness, hypotonia | HC (3 m), S (9 m), W (1.5 y) | No | Na | No | Na | Na | Na | No | na | Normal intellect/ no seizure | 1978 (2 y) | + | Na | Na | Na |  |
| P23/M/2.0 y | Birth | Muscle weakness, hypotonia, FD, RD | HC (3 m), S (1 y) | No | Elbow, hip | No | RD at birth | Na | Na | FD at birth | No | Normal intellect/ no seizure | 1200 (1 y) | + | Na | WMH | Na | Mechanical ventilation at birth, pectus excavatum |
| P24/M/2.0 y | Birth | Muscle weakness, hypotonia, weak cry | HC (1 y), S (1 y) | No | Knee, ankle | No | Na | Na | Na | No | Na | Intellectual delay/ no seizure | 1246 (1 y) | +, MNCV reduced | ± (2 y) | WMH, OP (1.8 y) | Na | Pectus carinatum |
| P25/M/2.5 y | Birth | Muscle weakness, hypotonia weak cry, FD | Can’t raise head | No | Knee | No | RRI, severe pneumonia | Na | Normal | FD at birth | Na | Normal intellect/ no seizure | 4404 (2 m) | MNCV reduced | Na | Normal (2 m) | Na | Died at 2.5 y following severe pneumonia, pectus excavatum |
| P26/M/2.7 y | Birth | Muscle weakness, hypotonia, weak cry | HC (7 m), S (10 m) | No | Knee, ankle | No | No | Na | Na | CD | Na | Normal intellect/ no seizure | 3148 (10 m) | Na | Na | WMH (6 m) | Na | Consanguineous parents, pectus carinatum |
| P27/M/2.8 y | Birth | Muscle weakness, hypotonia, weak cry, FD | HC (4 m), S (11 m) | No | Knee, elbow | Scoliosis (2.3 y) | RRI | Normal | PFO (1 m) | Constipation, CD | Yes | Normal intellect/ no seizure | 5430 (1 m) | Na | Na | Normal (1 m), WMH (17 m) | Na | Dislocation of hip, pectus excavatum |
| P28/M/3.1 y | Birth | Muscle weakness, hypotonia | HC (4 m), S (1.5 y) | No | Knee, ankle, elbow | No | No | Sinus tachycardia (2 m) | Na | No | Yes | Intellectual delay/ no seizure | 3385 (2 m) | Na | Na | Na | Na | Pectus carinatum |
| P29/M/3.1 y | 4 m | Muscle weakness, hypotonia | HC (1 y), S (7 m) | No | Knee | No | No | Na | Na | No | Yes | Normal intellect/ no seizure | 2874 (7 m) | + (7 m) | Na | WMH (7 m, 1.1 y) | Diffuse fatty infiltration | Pectus carinatum |
| P30/F/3.1 y | Birth | Muscle weakness, hypotonia | S (1 y) | No | Knee | No | RRI (2 y) | Na | Na | No | Yes | Normal intellect/ no seizure | 2667 (1 y) | + | − (11 m) | WMH (6 m, 1 y) | Na |  |
| P31/M/3.3 y | Birth | Muscle weakness, hypotonia | HC (9 m), S (9 m) | No | Knee | No | No | Sinus arrhythmia (6 m), normal (1.4 y) | Mild TR (6 m), Normal (1.4 y) | No | Yes | Normal intellect/ no seizure | 3670 (6 m) | + (6 m) | Na | WMH (6 m) | Na |  |
| P32/F/3.4 y | Birth | Muscle weakness, hypotonia, FD | HC (12 m), S (14 m) | No | Knee, ankle | No | RRI, severe pneumonia | Sinus tachycardia | Normal | FD at birth | Na | Normal intellect/ no seizure | 1630 (1.4 y) | +, MNCV and MNCMAPA reduced | Na | WMH | Na | Died at 3.4 y following severe pneumonia |
| P33/M/3.5 y | 4 m | Muscle weakness, hypotonia | S (10 m) | No | Knee, ankle | No | No | Normal | Normal | No | Yes | Normal intellect/ febrile seizure (2.3 y) | 2431 (10 m) | Na | Na | WMH (1.9 y) | Na |  |
| P34/F/3.5 y | Birth | Muscle weakness, hypotonia weak cry | HC (18 m), S (1 y) | No | No | No | RRI | Na | Na | Constipation, CD | Yes | Normal intellect/ no seizure | 3230 (2 m) | + (3 m) | Na | Na | Na | Pectus carinatum |
| P35/M/3.8 y | Birth | Muscle weakness, hypotonia, weak cry | S (7 m) | No | Knee | No | RRI | Normal | Normal | CD, constipation | Yes | Normal intellect/ no seizure | 2465 (6 m) | Na | Na | WMH, PH (2 y) | Diffuse fatty infiltration | Dislocation of hip |
| P36/M/3.9 y | Birth | Muscle weakness, hypotonia | S (1.5 y) | No | Knee | No | Na | Na | Na | Constipation | Na | Normal intellect/ febrile seizure (3 y) | Na | + | Na | WMH | Na | Arab, consanguineous parents, Case 9’s brother |
| P37/M/4.0 y | Birth | Muscle weakness, hypotonia, weak cry, FD | Can’t raise head | Na | Na | Na | RRI | Na | Na | FD at birth | Na | Normal intellect/ no seizure | 1552 (5 m) | Na | Na | WMH | Na | Died at 4 y following severe pneumonia |
| P38/M/4.2 y | Birth | Muscle weakness, hypotonia, weak cry, FD | HC (31 m), S (6 m) | No | Knee, ankle | Lordosis (3 y) | No | Na | Na | Constipation | Yes | Normal intellect/ no seizure | 3483 (3 m) | + (5 m) | Na | Normal (1 m), WMH (6 m) | Na |  |
| P39/M/4.3 y | Birth | Muscle weakness, hypotonia, weak cry, FD | HC (3 y), S (17 m) | No | Knee | No | RRI (2-4 y) | Normal | PFO (6 m) | CD, constipation | Yes | Normal intellect/ no seizure | Na | Normal (6 m) | Na | WMH (5 m) | Na |  |
| P40/F/4.6 y | Birth | Muscle weakness, hypotonia | HC (1.5 y), S (8 m) | No | No | No | No | Normal | Normal | No | No | Intellectual delay/ no seizure | 2000  (1.2 y) | + (8 m) | Na | WMH (7 m) | Na |  |
| P41/M/4.7 y | Birth | Muscle weakness, hypotonia, FD | S (10 m) | No | Knee | No | No | Na | Na | FD at birth | Yes | Normal intellect/ no seizure | 6000 (birth) | + (11 m) | Na | WMH (11 m) | Na | Pectus carinatum |
| P42/M/4.8 y | Birth | Muscle weakness, hypotonia, weak cry, FD | HC (2 y), S (2 y) | No | Knee, ankle, elbow, hip | No | No | Normal | PFO (4 m) | CD | No | Normal intellect/ no seizure | 3496 (1.3 y) | + (4 m) | Na | WMH (14 m) | Diffuse fatty infiltration | Pectus carinatum |
| P43/M/5 y | Birth | Muscle weakness, hypotonia, weak cry | S (1 y) | No | Knee, ankle, elbow | No | No | Na | Normal | CD | Yes | Normal intellect/ no seizure | 1878 (9 m) | + | Na | Normal (6 d) | Diffuse fatty infiltration | Dislocation of hip |
| P44/F/5.1 y | Birth | Muscle weakness, hypotonia | Can’t raise head | No | Knee, elbow, ankle | No | RRI (3-4 y) | Na | Na | CD | No | Normal intellect/ no seizure | 8010 (2 m) | Na | Na | Normal (5 m) | Na | Pectus carinatum |
| P45/F/5.1 y | 5 m | Muscle weakness, hypotonia | HC (5 m), S (8 m) | No | Ankle | No | No | Normal | Normal | CD | Yes | Normal intellect/ no seizure | 88680 (birth) | MNCV and MNCMAPA reduced (5 m) | Na | WMH (5 m) | Na | Case 15’s elder sister, pectus carinatum |
| P46/F/5.3 y | Birth | Muscle weakness, hypotonia, RD | S (18 m) | No | Knee, ankle, elbow | No | RRI (4 y) | Normal | Normal | CD, constipation | Yes | Normal intellect/ no seizure | 86425 (birth) | Na | Na | WMH (4 m) | Na | Pectus carinatum |
| P47/M/5.5 y | Birth | Muscle weakness, hypotonia | HC (10 m), S (8 m) | No | Knee, elbow | No | RRI (4 y) | Normal | Na | Constipation | Yes | Normal intellect/ no seizure | 3488 (5 m) | + | Na | WMH, PH (6 m, 1 y) | Na |  |
| P48/F/5.6 y | Birth | Muscle weakness, hypotonia, weak cry | HC (7 m), S (8 m) | No | Knee, elbow | Scoliosis (5 y) | No | Na | Na | No | Yes | Normal intellect/ no seizure | 924 (5.2 y) | Na | Na | WMH, PH (11 m, 3.8 y) | Na |  |
| P49/M/5.7 y | Birth | Muscle weakness, hypotonia, weak cry, FD | HC (6 m), S (1 y) | Loss of rolling (3.8 y) | Knee, ankle | Lordosis (4 y) | No | Normal | PFO (1 m), Normal (11 m) | CD, constipation | No | Normal intellect/ no seizure | 10661 (1 m) | + (1 m) | Na | Focal changes (3 m) | Na | Pectus excavatum, dislocation of hip |
| P50/M/5.8 y | Birth | Muscle weakness, hypotonia | HC (6 m), S (9 m) | No | Knee | No | No | Normal | PFO (1 y), Normal (3 y) | Constipation, CD | Yes | Normal intellect/ no seizure | 2151 (5 m) | + (6 m) | Na | WMH (5 m) | Na |  |
| P51/F/6 y | Birth | Muscle weakness, hypotonia | HC (8 m), S (1 y), W (2.5 y) | No | Ankle | No | RRI (4 y) | Normal | Normal | No | Yes | Normal intellect/ no seizure | 2500 (4.7 y) | + (1.4 y) | Na | WMH (4.1 y) | Na |  |
| P52/F/6 y | Birth | Muscle weakness, hypotonia, weak cry | HC (2 m), S (7 m) | Loss of S (4.1 y) | Knee, elbow, ankle | Scoliosis (6 y) | RRI (4 y) | Na | Na | CD | No | Normal intellect/ no seizure | 2757 (6 m) | Na | Na | WMH, PH (6 m) | Na |  |
| P53/M/6 y | 4 m | Muscle weakness, hypotonia | HC (5 m), S (6 m), W (3.5 y) | No | Knee | No | No | Na | Normal | No | Yes | Normal intellect/ no seizure | 1701 (1.4 y) | + (7 m) | Na | WMH (10 m, 3.4 y) | Diffuse fatty infiltration |  |
| P54/M/6 y | Birth | Muscle weakness, hypotonia, RD | HC (2 y), S (1 y) | No | No | No | RD at birth | Na | LVFT | CD | Yes | Normal intellect/ no seizure | 43830 (birth) | + | Na | Normal (1 m), WMH (9 m) | Na | Mechanical ventilation at birth, dislocation of hip, pectus excavatum |
| P55/M/6 y | Birth | Muscle weakness, hypotonia, FD, RD | HC (3 y), S (3 y) | No | Knee, elbow, ankle, hip | No | RD at birth, RRI (1-4 y) | Na | Na | CD, SD, constipation | Yes | Normal intellect/ no seizure | 88680 (birth) | MNCV and MNCMAPA reduced (17 m) | − (6 m) | WMH (1 y) | Na | Mechanical ventilation at birth, pectus excavatum |
| P56/M/6.0 y | Birth | Muscle weakness, hypotonia, weak cry, FD | S (9 m) | Na | Yes | Scoliosis (6 y) | Na | Na | Na | FD at birth | No | Normal intellect/ no seizure | 491 (6 y) | + | Na | WMH | Na | Case 8’s elder brother |
| P57/M/6.3 y | Birth | Muscle weakness, hypotonia | HC (6 m), S (8 m) | No | Knee, elbow, hip | Scoliosis (6 y) | RRI, severe pneumonia (6 y) | Normal | Normal | No | No | Normal intellect/ no seizure | 91 (6 y) | +, MNCV reduced | Na | WMH (6.1 y) | Na | Consanguineous parents, died at 6.3 y following severe pneumonia |
| P58/F/6.3 y | Birth | Muscle weakness, hypotonia | HC (5 m), S (6 m), W (2 y) | Loss of W (6 y) | Ankle | No | RRI (4-6 y) | Na | Na | CD, constipation | Yes | Normal intellect/ no seizure | 3551 (8 m) | Na | − (1 y) | WMH | Na |  |
| P59/M/6.5 y | Birth | Muscle weakness, hypotonia, weak cry, FD | HC (4 m), S (6 m) | No | Knee, ankle | No | RRI (1-2 y) | Normal | PFO (5.3 y) | FD at birth | Yes | Normal intellect/ no seizure | 3714 (5 m) | +, MNCMAPA reduced (9 m) | Na | WMH (5 m) | Na | Dislocation of hip (3 y) |
| P60/M/6.7 y | 4 m | Muscle weakness | HC (4 m), S (6 m), W (1.5 y) | No | Elbow | No | RRI (3-4 y) | Na | Na | No | No | Normal intellect/ no seizure | 2000 (3 y) | Na | Na | Na | Diffuse fatty infiltration |  |
| P61/F/6.9 y | Birth | Muscle weakness, hypotonia | HC (4 m), S (7 m) | No | Knee, ankle | No | RRI (3 y) | Normal | Normal | CD | Yes | Normal intellect/ no seizure | 1715 (11 m) | + (11 m) | Na | WMH (10 m) | Na |  |
| P62/M/7.2 y | Birth | Muscle weakness, hypotonia, weak cry, FD | S (2.5 y) | No | Yes | Scoliosis (6 y) | RRI | Na | PFO (5 m) | CD | No | Normal intellect/ no seizure | 2612 (3 m) | + | Na | Normal (3 m), WMH (7 m) | Na | Died at 7.2 y following severe pneumonia |
| P63/M/7.5 y | Birth | Muscle weakness, hypotonia, weak cry, FD | HC (10 m), S (1 y) | Loss of S (5.6 y) | Knee, ankle, elbow, hip | Lordosis (2 y) | RRI (1-6 y) | Na | Mild VSH and TR | CD, constipation | No | Normal intellect/ no seizure | 2167 (7 m) | + (10 m) | Na | WMH (7 m) | Na | Pectus carinatum |
| P64/M/7.8 y | Birth | Muscle weakness, hypotonia, weak cry | HC (3 m), S (7 m) | No | Knee, ankle, elbow, hip | No | RRI | Na | Normal | CD, constipation | Yes | Normal intellect/ no seizure | 1662 (1.8 y) | Na | Na | WMH, PH (1.8 y) | Na |  |
| P65/F/8.0 y | Birth | Muscle weakness, hypotonia | Can’t raise head | No | Knee, ankle, elbow | Scoliosis (4 y) | RRI (1-6 y) | Sinus tachycardia (5 m) | Na | CD | No | Normal intellect/ no seizure | 45243 (birth) | Na | Na | Normal (7 d), WMH (6 m) | Na | Pectus excavatum |
| P66/M/8.1 y | Birth | Muscle weakness, hypotonia | S (7 m) | No | Knee, ankle, elbow, hip | Scoliosis (6 y) | No | Na | Na | CD, SD, constipation | Yes | Normal intellect/ no seizure | 611 (2 y) | +, MNCV and MNCMAPA reduced (2 y) | − (2 y) | WMH | Diffuse fatty infiltration |  |
| P67/F/8.3 y | Birth | Muscle weakness, hypotonia, weak cry | HC (5 m), S (1.5 y), W (6 y) | No | Knee, ankle, hip | No | No | Normal | Normal | CD, constipation | Yes | Normal intellect/ no seizure | 4627 (9 m) | Na | Na | WMH (9 m) | Diffuse fatty infiltration |  |
| P68/M/8.3 y | Birth | Muscle weakness, hypotonia, weak cry | HC (1 y), S (2 y) | No | Knee, ankle, elbow | Lordosis (3 y) | RRI (3 y) | Na | Mild pulmonary regurgitation (6 m) | No | Yes | Normal intellect/ no seizure | 10080 (birth) | + | Na | WMH  (6 m) | Diffuse fatty infiltration | Pectus carinatum |
| P69/M/8.3 y | Birth | Muscle weakness, hypotonia | HC (7 m), S (1.2 y) | Muscle weakness (6.4 y) | Knee, ankle, elbow | Scoliosis (6 y) | RRI | Na | Na | CD | No | Normal intellect/ no seizure | 1019 (5 y) | + | Na | WMH, OP (1.9 y) | Na | Dislocation of hip |
| P70/M/8.5 y | Birth | Muscle weakness, hypotonia, weak cry, RD | S (2 y) | Muscle weakness (6.6 y) | Knee, elbow | No | RD at birth, RRI (1-3 y) | Normal | Na | No | Yes | Normal intellect/ no seizure | 2798 (11 m) | Na | Na | Na | Na |  |
| P71/M/8.6 y | Birth | Muscle weakness, hypotonia, weak cry, FD | HC (1 y), S (1.1 y) | Loss of S (6.5 y) | Knee, ankle, elbow, hip | Scoliosis (5 y) | RRI | Normal | MTR (2 y) | CD, constipation | Yes | Normal intellect/ no seizure | 3983 (3 m) | +, MNCV reduced (2 m) | − (3 m) | WMH, PH (9 m, 2.3 y) | Na | Case 8’s younger brother, died at 8.6 y following severe pneumonia |
| P72/M/8.7 y | Birth | Muscle weakness, hypotonia, weak cry, FD, RD | HC (3 y) | No | Knee, ankle, elbow, hip | Scoliosis (1 y) | RD at birth, RRI  (1-3 y) | Na | Normal | No | No | Normal intellect/ no seizure | 5901 (1.1 y) | + (4 m) | Na | WMH | Na | Mechanical ventilation at birth, pectus excavatum |
| P73/M/8.7 y | Birth | Muscle weakness, hypotonia, weak cry | HC (1 y), S (1.5 y) | No | Knee, ankle, elbow | Scoliosis (4 y) | RRI | Normal | Normal | Na | No | Normal intellect/ no seizure | 1200 (2 y) | +, MNCV reduced | Na | WMH | Na | Consanguineous parents, pectus excavatum |
| P74/M/8.8 y | 3 m | Muscle weakness, hypotonia | HC (4 m), S (8 m) | No | Knee, elbow, hip | Scoliosis (6 y) | RRI | Normal | Normal | Constipation | No | Normal intellect/ no seizure | 1150 (1.5 y) | + | − (2.7 y) | WMH, OP (9 m, 2 y) | Na | Pectus carinatum |
| P75/F/8.8 y | 4 m | Muscle weakness, hypotonia, weak cry | HC (6 m), S (11 m) | Loss of HC (6.8 y), rolling (5 y) | Knee, ankle, elbow, hip | Scoliosis (7 y) | RRI (3 y) | Normal | Increased left ventricle (1 y) | CD | No | Normal intellect/ no seizure | 1730 (6 m) | Normal (6 m) | Na | WMH (1.3 y) | Diffuse fatty infiltration |  |
| P76/F/9 y | Birth | Muscle weakness, hypotonia, weak cry | HC (4 m), S (7 m) | Muscle weakness (7 y) | Knee | No | RRI (7 y) | Na | Na | Constipation | Yes | Intellectual delay/ no seizure | 4224 (8 m) | + | Na | WMH, OP, PH (2 y) | Na |  |
| P77/F/9.3 y | 3 m | Muscle weakness, hypotonia, weak cry | HC (5 m), S (8 m), W (3 y) | Loss of W (8.5 y) | Knee, ankle | Lordosis (3 y) | RRI | Na | Na | FD at birth | No | Normal intellect/ no seizure | 2025 (10 m) | + | Na | WMH (10 m) | Na |  |
| P78/M/9.3 y | Birth | Muscle weakness, hypotonia | HC (6 m), S (9 m) | Loss of S (5 y) | Knee, elbow | No | RD (7.3 y),  RRI (7 y) | Na | Na | CD | Yes | Normal intellect/ no seizure | 8111  (1.2 y) | Na | − (2 y) | WMH (1.8 y) | Diffuse fatty infiltration, atrophy | Case 19’s cousin |
| P79/M/9.3 y | Birth | Muscle weakness, hypotonia, weak cry | HC (4 m), S (1 y) | Loss of rolling (4 y) | Knee, ankle, elbow | Scoliosis (6 y) | RRI (8 y) | na | Normal | CD, constipation | Yes | Normal intellect/ no seizure | 1778 (1 y) | + (6 m) | − (10 m) | WMH, OP (8 m) | Na | Pectus carinatum |
| P80/F/9.4 y | 3 m | Muscle weakness, hypotonia, weak cry | HC (3 m), S (8 m) | Loss of S (8.5 y) | Knee, ankle, elbow | Scoliosis (7 y) | RRI (7 y) | Sinus tachycardia (1.8 y), ST-T wave change (3.7 y) | LVEF with 45% (1.8 y), normal (4 y) | No | No | Normal intellect/ no seizure | 2540 (7 m) | + (6 m) | Na | WMH (6 m) | Na | Consanguineous parents, pectus carinatum |
| P81/M/9.4 y | Birth | Muscle weakness, hypotonia, weak cry, FD | HC (7 m), S (1 y), W (3 y) | No | Knee, ankle | Lordosis (6 y) | No | Normal | Normal | Constipation | Yes | Normal intellect/ no seizure | 1800 (8 y) | + | Na | WMH (8 y) | Na | Case 51’s brother |
| P82/M/9.5 y | Birth | Muscle weakness, hypotonia | HC (8 m), S (10 m) | No | Knee, elbow | No | RRI | Normal | Normal | CD | Yes | Normal intellect/ epilepsy (9 y) | 3436 (11 m) | + | ± (1.2 y) | Normal (3 m), WMH (5, 11 m) | Na |  |
| P83/F/9.7 y | Birth | Muscle weakness, hypotonia, FD | HC (6 m), S (9 m) | No | Knee, ankle, elbow | Scoliosis (5 y) | RRI | Na | Na | CD | Yes | Normal intellect/ no seizure | 1222 (8 m) | + (10 m) | Na | WMH (9 y) | Na |  |
| P84/M/9.8 y | Birth | Muscle weakness, hypotonia, RD | HC (5 m), S (9 m) | No | Knee, ankle, elbow | Scoliosis (6 y) | RD at birth, RRI (8-9 y) | Normal | Mild MR, TR (8.3 y) | CD | No | Normal intellect/ no seizure | 17000 (birth) | + | Na | WMH | Na | Died at 9.8 y |
| P85/M/10.3 y | Birth | Muscle weakness, hypotonia, FD | HC (3 m), S (7 m) | No | Knee, elbow | Scoliosis (7 y) | RRI | Sinus arrhythmia (1 y), normal (5 y) | Na | CD, constipation | No | Normal intellect/ no seizure | 4089 (2 m) | + | Na | WMH (1 y, 3.2 y) | Na |  |
| P86/F/10.5 y | Birth | Muscle weakness, hypotonia | HC (6 m), S (2 y) | Loss of S (8 y) | Knee, elbow, hip | Scoliosis (9 y) | No | Na | Normal | CD | No | Normal intellect/ no seizure | 1500 (2 y) | + | Na | WMH | Na | Dislocation of hip |
| P87/M/10.6 y | Birth | Muscle weakness, hypotonia, weak cry, FD | HC (1 y), S (2 y) | Loss of S (6 y) | Knee, ankle, elbow, hip | Scoliosis (6 y) | RD from 6y, RRI | Sinus arrhythmia | Normal | CD, constipation | Yes | Normal intellect/ no seizure | 6045 (3 m) | +, MNCV reduced (1.3 y) | Na | WMH (1.2 y) | Na | Invasive mechanical ventilation from 8.2 y, pectus carinatum |
| P88/M/10.8 y | 4 m | Muscle weakness, hypotonia | HC (4 m), S (10m), W (1.7 y) | No | No | No | No | Normal | Mild TR | No | Yes | Normal intellect/ no seizure | 343 (3 y) | + | − (2.4 y) | WMH (2y) | Diffuse fatty infiltration, atrophy | Pectus carinatum |
| P89/F/11.2 y | 3 m | Muscle weakness, hypotonia | HC (5 m), S (8 m), W (2 y) | Loss of W (8 y) | Knee, ankle, elbow | Scoliosis (8 y) | RRI (5-11 y) | No | No | No | No | Normal intellect/ no seizure | 900 (6 y) | + (5.6 y) | Na | WMH (2 y) | Na | Case 60’s elder sister |
| P90/F/11.7 y | 5 m | Muscle weakness, hypotonia | HC (5 m), S (10m), W (1.7 y) | No | Ankle | Lordosis (3 y) | No | Na | Na | No | No | Normal intellect/ no seizure | 368 (4 y) | Na | Na | WMH | Na | Pectus carinatum |
| P91/M/11.7 y | Birth | Muscle weakness, hypotonia, weak cry, FD | HC (6 m), S (1 y) | Loss of HC (9.8 y) | Knee, ankle, elbow | Scoliosis (9 y) | No | Na | Na | CD | No | Normal intellect/ no seizure | 852 (6 m) | + (6 m) | Na | WMH (2y) | Na | Pectus carinatum |
| P92/M/11.8 y | Birth | Muscle weakness, hypotonia, FD | HC (8 m), S (14 m), W (4 y) | Loss of W (11 y) | Knee, ankle, elbow, hip | Spinal ankylosis (6 y) | No | Na | Na | FD at birth, CD | No | Normal intellect/ no seizure | 1899 (1.3 y) | + | Na | WMH, OP (2y) | Na |  |
| P93/M/12.6 y | Birth | Muscle weakness, hypotonia, RD | HC (8 m), S (1 y) | Loss of rolling (12 y) | Elbow | No | RD at birth | Na | Na | CD, constipation | Yes | Normal intellect/ no seizure | 2481 (8 m) | +, MNCV and MNCMAPA reduced | Na | WMH (2y) | Na | Dislocation of hip |
| P94/F/12.7 y | Birth | Muscle weakness, hypotonia, FD | HC (1 y), S (1.3 y) | Loss of HC (11 y), S (11 y) | Knee, ankle, elbow, hip | Scoliosis (3 y) | RRI, RD (11 y) | Na | Na | CD | No | Normal intellect/ no seizure | 3264 (10 m) | +, MNCV and MNCMAPA reduced (10 m) | − (2 y) | WMH (10 m) | Na | Non-invasive mechanical ventilation from 12.6 y, died at 12.7 y following severe pneumonia; pectus excavatum |
| P95/F/12.8 y | 4 m | Hypotonia, weak cry | HC (3 m), S (10 m), W (4.5 y) | Loss of S (7.5 y), W (6.8 y) | Knee, elbow | Scoliosis (8 y) | RD (10.8 y) | Normal | Na | Constipation | No | Normal intellect/ no seizure | 2427 (9 m) | + | Na | WMH | Na | Uighur, pectus carinatum |
| P96/F/13 y | Birth | Muscle weakness, hypotonia | HC (2 y), S (1 y) | No | Knee, ankle, elbow | Scoliosis (9 y) | No | Na | Na | No | No | Normal intellect/ no seizure | 3008 (3 y) | Na | Na | WMH (2.8 y) | Na |  |
| P97/F/13 y | Birth | Muscle weakness, hypotonia | S (10 m) | Na | Knee, ankle, elbow | Scoliosis (8 y) | RRI, severe pneumonia | Na | Na | Na | Na | Normal intellect/ no seizure | Na | Na | Na | WMH | Na | Uighur, Case 95’s elder sister, died at 13 y following severe pneumonia |
| P98/M/13 y | Birth | Muscle weakness, hypotonia, weak cry, FD | HC (8 m), S (1 y) | No | Ankle, elbow | Scoliosis (12 y) | No | Normal | Na | SD (8 y), constipation | Na | Intellectual regression/ epilepsy (3 y) | Na | + (3.8 y) | Na | WMH, OP (2y, 3.4y) | Na | Died at 13 y, following epilepsy |
| P99/M/13 y | Birth | Muscle weakness, hypotonia | HC (3 m), S (1 y) | Loss of rolling (12 y) | Knee, ankle, elbow, hip | Lordosis (7 y) | RRI (2 y) | Normal | Na | CD | No | Normal intellect/ no seizure | 2762 (1 m) | + | − (6 m) | WMH (6 m) | Na | Pectus carinatum |
| P100/M/13.1 y | Birth | Muscle weakness, hypotonia, weak cry, FD | HC (7 m), S (9 m), W (1.5 y) | Loss of W (1.7 y) | Knee, ankle, elbow, hip | Scoliosis (7 y) | RRI | Normal | Normal | CD, SD, constipation | No | Normal intellect/ neonatal epilepsy | 1011 (3.3 y) | + | − (3 y) | WMH, PH (4.3 y) | Na | Died at 13.1 y following severe pneumonia |
| P101/M/13.2 y | Birth | Muscle weakness, hypotonia, weak cry | HC (1 y), S (1.5 y) | No | Knee, elbow | Scoliosis (8 y) | RD from 11 y | Na | Normal | FD at birth, CD | Yes | Normal intellect/ no seizure | 1261 (1 y) | + | Na | WMH | Na | Non-invasive mechanical ventilation from 11.2 y, pectus carinatum |
| P102/F/13.5 y | Birth | Muscle weakness, hypotonia | HC (13 m), S (1 y) | Loss of S (9 y) | Knee, ankle, elbow, hip | Scoliosis (8 y) | RRI | Sinus tachycardia (8 days) | PFO | CD, SD, constipation | No | Normal intellect/ epilepsy (3y) | 1573 (1.5 y) | +, MNCV and MNCMAPA reduced (4 y) | − (1.3 y) | Normal (15 d), WMH, OP (1.7 y) | Na | Died at 13.5 y following FD |
| P103/M/13.6 y | 4 m | Muscle weakness, hypotonia | HC (4 m), S (9 m) | Loss of HC (10 y) | Knee, ankle, elbow, hip | Scoliosis (6 y) | RRI (12 y) | Na | Na | FD at birth, CD | No | Normal intellect/ no seizure | 4436 (10 m) | + | − (3.5 y) | WMH | Na | Dislocation of hip |
| P104/F/13.8 y | 3 m | Muscle weakness, hypotonia, weak cry | HC (6 m), S (10 m) | Loss of rolling (6 y), S (9 y) | Knee, ankle, elbow, hip | Scoliosis (3 y) | RRI (1-12 y) | Normal | Normal | FD at birth, CD | No | Normal intellect/ no seizure | 596 (1 y) | + | − (1.8 y) | WMH (1.3 y) | Na | Case 18’s sister, pectus excavatum |
| P105/F/13.9 y | 3 m | Muscle weakness, hypotonia, weak cry | HC (5 m), S (10 m) | Loss of rolling, HC, S (7 y) | Knee, ankle, elbow | Scoliosis (6 y) | RD from12 y | Na | Na | CD, SD | No | Normal intellect/ no seizure | 3620 (6 m) | + | Na | WMH (2.3 y, 2.7 y) | Na | Case 64’s sister |
| P106/M/14 y | Birth | Muscle weakness, hypotonia | HC (3 m), S (7 m) | Loss of S (8 y), HC (10 y) | Knee, ankle, elbow | Scoliosis (8 y) | RD from 12 y | Na | Na | CD | No | Normal intellect/ no seizure | Na | Na | Na | WMH | Na | Case 52’s brother |
| P107/M/14.3 y | Birth | Muscle weakness, hypotonia | HC (1 y), S (1 y) | Loss of rolling, HC (7 y), S (9 y) | Knee, ankle, elbow, hip | Scoliosis (11 y) | RRI | Normal | Na | CD, SD | No | Normal intellect/ no seizure | 259 (11.1 y) | Na | Na | Na | Diffuse fatty infiltration, atrophy | Died at 14.3 y following severe pneumonia |
| P108/M/15 y | Birth | Muscle weakness, hypotonia | HC (10 m), S (14 m) | No | Knee, ankle, elbow | Scoliosis  (8 y) | RD (14 y), RRI | Na | Na | SD, constipation | No | Normal intellect/ epilepsy (13y) | 770 (3 m) | + | − (3 y) | WMH | Na | Died at 15.0 y following epilepsy |
| P109/M/15.5y | 6 m | Muscle weakness, hypotonia | HC (3 m), S (1 y) | No | Knee, ankle, elbow | Scoliosis (10 y) | RRI | Normal | Normal | CD | No | Intellectual delay/ epilepsy (13y) | Na | Na | Na | Na | Na | Case 40’s elder brother, died at 15.5 y following severe pneumonia |
| P110/F/17.1 y | 4 m | Muscle weakness, hypotonia | HC (5 m), S (1 y), W (7 y) | No | Knee, elbow | Scoliosis (4 y) | RD (16 y) | Normal | Na | No | No | Normal intellect/ febrile seizure (4y), epilepsy (15.5 y) | 1565 (6 m) | + | Na | WMH (6 m, 4 y) | Na | Newborn subarachnoid hemorrhage |
| P111/M/17.3 y | Birth | Muscle weakness, hypotonia | S (1 y) | Loss of S (14 y) | Knee, ankle, elbow, hip | Scoliosis (0.5 y) | RD (8 y) | Na | Mild tricuspid insufficiency | CD, SD, constipation | No | Normal intellect/ no seizure | 3549 (6 m) | + (6 m) | − (6 m) | WMH (3 y, 8 y) | Diffuse fatty infiltration |  |
| P112/M/18 y | Birth | RD | HC (6 m), S (1.1 y) | Na | Knee, ankle, elbow, hip | Scoliosis (12 y) | RD at birth | Na | Na | CD, SD, constipation | No | Intellectual delay/ no seizure | 9640 (8 m) | + (6 m) | − (1.3 y) | WMH (2y) | Na | Died at 18 y following FD |
| P113/F/18.6 y | 2 m | Muscle weakness, hypotonia | S (7 m), W (4 y) | Loss of S (11 y), W (8 y) | Knee, ankle, elbow, hip | Scoliosis (6 y) | No | Na | Na | CD, SD, constipation | No | Normal intellect/ no seizure | 1097 (1.2 y) | + | − (1.2 y) | WMH (13 m) | Diffuse fatty infiltration |  |
| P114/F/20.8 y | Birth | Muscle weakness, hypotonia | HC (5 m), S (9 m) | Loss of rolling, (6 y), S (19 y) | Knee, ankle, elbow, hip | Scoliosis (8 y) | RD (13 y), RRI (13-20 y) | Sinus tachycardia (10 y) | Mild TR | CD | No | Normal intellect/ no seizure | 4479 (3 m) | + (6 m, 8.8 y) | Na | WMH (8.8 y, 11.1 y) | Na | Non-invasive mechanical ventilation from 18.9 y |
| P115/M/26 y | 4 m | Muscle weakness, hypotonia | HC (5 m), S (11 m), W (2 y) | Loss of W (5 y) | Knee, ankle, elbow | Scoliosis (6 y) | RD (24 y) | Na | Na | No | Yes | Normal intellect/ epilepsy (15 y) | 356 (6 y) | + | ± (13.5 y) | WMH | Na |  |
| P116/M/ 27.3 y | 3 m | Muscle weakness, hypotonia | HC (5 m), S (8 m), W (1.5 y) | Loss of W (8 y) | Knee, ankle, elbow | Scoliosis, lordosis (6 y) | RRI (13-15 y) | Na | Na | CD, constipation | No | Intellectual regression/ epilepsy (13 y) | 1000 (5 y) | + | Na | WMH (13.9 y) | Na | Intellectual regression after epilepsy |
| P117/M/3.2 y | 1.2 y | myopathic gait | HC (3 m), S (7 m), W (1.2 y) | No | No | No | No | Normal | Normal | No | Yes | Normal intellect/ no seizure | 1344 (2 y) | MNCV and MNCMAPA reduced (1 y) | Na | WMH (2 y) | Diffuse fatty infiltration |  |
| P118/F/5.5 y | 2.5 y | difficulty running and jumping | HC (3 m), S (6 m), W (1.1 y), R (2 y) | No | No | No | RRI (3-4 y) | Sinus tachycardia | PFO | Constipation | Yes | Normal intellect/ febrile seizure (1.5 y ) | 2253 (11 m) | Na | Na | WMH (1.8 y) | Na |  |
| P119/M/6 y | 1.5 y | myopathic gait | HC (3 m), S (6 m), W (1.5 y), R (4 y) | No | No | No | No | Normal | Normal | No | Yes | Normal intellect/ no seizure | 4362 (3.2 y) | + (3.3 y) | Na | WMH | Na |  |
| P120/M/6.3 y | 1.1 y | myopathic gait | HC (2 m), S (6 m), W (1.1 y), R (4 y) | No | No | No | RRI (1-4 y) | Sinus arrhythmia | Na | No | No | Normal intellect/ no seizure | 709 (8 m) | + | Na | WMH (3.6 y) | Na |  |
| P121/F/6.4 y | 1.5 y | myopathic gait | HC (3 m), S (6 m), W (1.5 y), R (4.5 y) | No | No | No | RRI | Normal | Na | No | Yes | Normal intellect/ no seizure | 1337 (2 y) | +, MNCV reduced (1.7 y) | Na | WMH (1.7 y) | Diffuse fatty infiltration |  |
| P122/M/6.4 y | 1.5 y | myopathic gait | HC (3 m), S (8 m), W (1.6 y), R (2.5 y) | No | No | No | No | Na | Na | No | Yes | Normal intellect/ no seizure | 2000 (5 y) | Normal (2.3 y) | ± (2.3 y) | Frontal horn of lateral ventrical (3.6 y) | Diffuse fatty infiltration |  |
| P123/F/7.9 y | 2 y | myopathic gait | HC (3 m), S (6 m), W (1.7 y), R (2.5 y) | Loss of W (5.5 y) | Knee, ankle | No | No | Sinus tachycardia | Na | No | Yes | Normal intellect/ febrile seizure (2.6 y) | 3078 (2.5) | +, MNCV and MNCMAPA reduced (1.6 y) | Na | WMH (1.6 y, 2.3 y) | Na | The grandfather's brother had epilepsy |
| P124/F/8.5 y | 1.5 y | myopathic gait | HC (4 m), S (6 m), W (1.5 y), R (3 y) | No | No | No | No | Na | Normal | Constipation | No | Normal intellect/ no seizure | 3103 (1 y) | + | Na | Na | Na |  |
| P125/F/9.6 y | 1.2 y | myopathic gait | HC (4 m), S (7 m), W (1.2 y), R (2.5 y) | No | No | No | No | Normal | Normal | No | No | Normal intellect/ no seizure | 1481 (1 y) | + | Na | Mild change (2.5 y, 8.9 y) | Na |  |
| P126/F/11.3 y | 2 y | myopathic gait | HC (3 m), S (6 m), W (1.5 y) | No | Ankle | Scoliosis (10 y) | RRI (1-7 y) | Sinus tachycardia | No | No | Yes | Normal intellect/ no seizure | 1018 (6 y) | +, MNCV reduced (6 y) | Na | WMH (5.7 y) | Diffuse fatty infiltration |  |
| P127/M/14.1 y | 6 y | difficulty running and jumping | HC (4 m), S (10 m), W (1.3 y) | No | No | No | No | Normal | Mild TR | No | No | Normal intellect/ no seizure | 2103 (6.4 y) | + | ± (7 y) | Mild change, OP (7.8 y) | Na | Case 125’s elder brother, died at 14.1 y |
| P128/F/18.2 y | 13 y | epilepsy | HC (4 m), S (6 m), W (1.5 y), R (17 y) | No | No | No | RRI (16 y) | Normal | Na | No | No | Normal intellect/ epilepsy (11 y) | 442 (13.2 y) | Na | Na | WMH, OP (13.2 y) | Na |  |
| P129/M/23.6 y | 1.3 y | myopathic gait | HC (3 m), S (6m), W (1.3 y), R (2 y) | No | No | No | No | Na | Mild TR | No | No | Normal intellect/ epilepsy (14 y) | 934 (15.5 y) | Na | Na | Posterior horn of lateral ventrical (18 y) | Na |  |
| P130/M/27 y | 2 y | difficulty running and jumping | HC (4 m), S (9 m), W (1.5 y) | No | No | No | No | Normal | Mild MR, TR | No | No | Normal intellect/ epilepsy (23 y) | 1025 (21 y) | Na | Na | WMH (22 y) | Na | Case 128’s elder brother |

CD: chewing difficulty; d: days; CK: creatine kinase; ECG: electrocardiogram; EMG: electromyogram; F: female; FD: feeding difficulty; HC: head control; LAFB: left anterior fascicular block; *LAMA2*-CMD: *LAMA2*-related congenital muscular dystrophy; LGMDR23: limb-girdle muscular dystrophy-23; LVEF: left ventricular ejection fraction; LVFT: left ventricular false tendon; M: male; m: months; MNCV: motor nerve conduction velocity; MNCMAPA: motor nerve compound muscle action potential amplitude; MR: mitral regurgitation; Na: not available; OP: occipital pachygyria; PFO: patent foramen ovale; PH: pontine hypoplasia; RD: respiratory difficulty; RRI: recurrent respiratory tract infection; S: sitting; SD: swallowing difficulty; TR: tricuspid regurgitation; UCG: ultrasonic cardiogram; VSH: ventricular septal hypertrophy; W: walking; WMH: abnormal white matter hyperintensities; y: years.
