# Supplementary figures and images for: Natural history and genetic study of LAMA2-related muscular dystrophy in a large Chinese cohort
Source: Orphanet J Rare Dis. 2021 Jul 19;16:319. doi: 10.1186/s13023-021-01950-x (PMC8287797; doi:10.1186/s13023-021-01950-x)

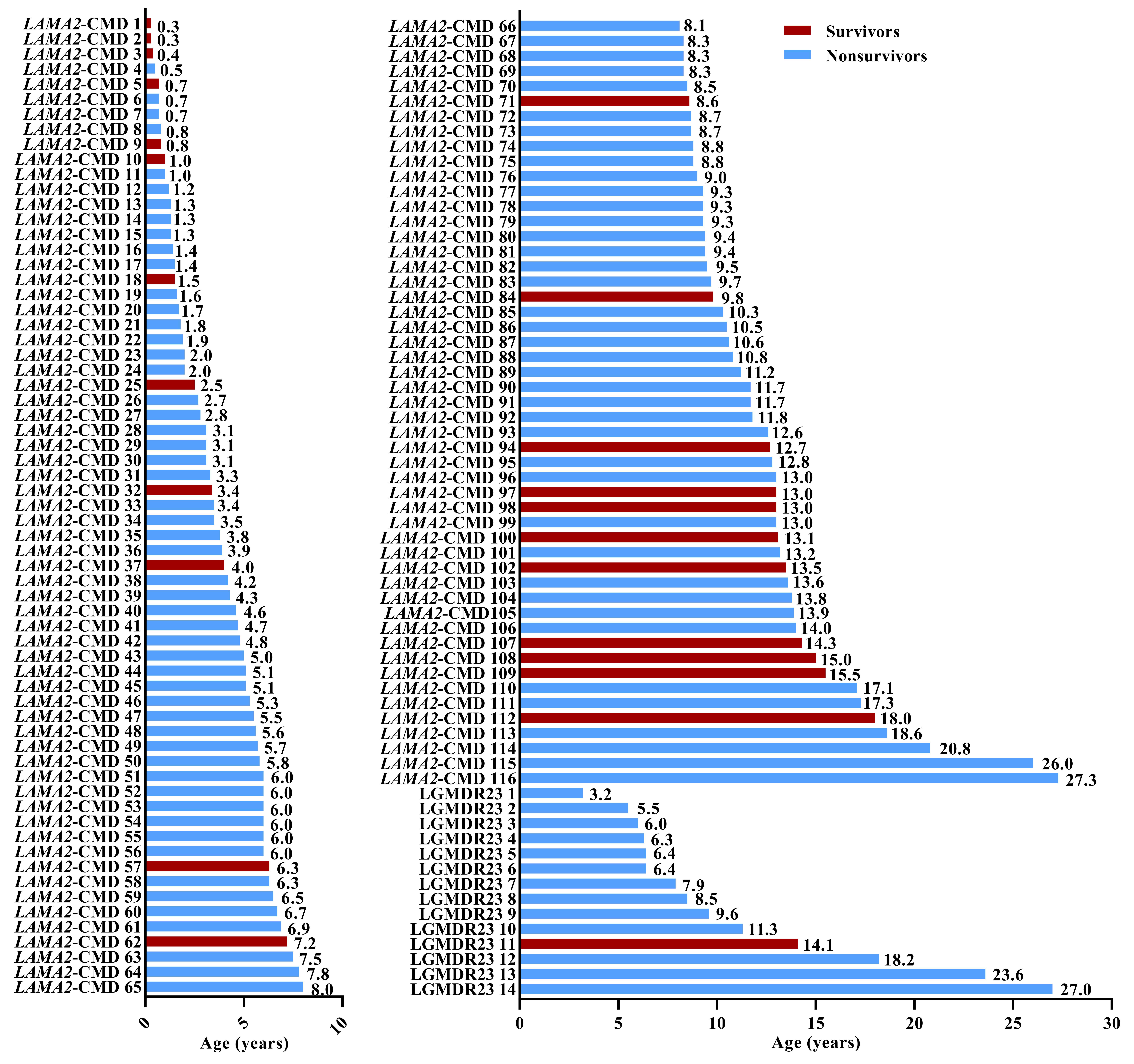

Supplement: Supplementary file 2 — Additional file 2. The ages of patients with LAMA2-related muscular dystrophy. [file 13023_2021_1950_MOESM2_ESM.tif]
