# Supplementary material for: Natural history and genetic study of LAMA2-related muscular dystrophy in a large Chinese cohort
Source: Orphanet J Rare Dis. 2021 Jul 19;16:319. doi: 10.1186/s13023-021-01950-x (PMC8287797; doi:10.1186/s13023-021-01950-x)
Supplement: Supplementary file 3 — Additional file 3. Effect of motor and epilepsy on survival of LAMA2-CMD. [file 13023_2021_1950_MOESM3_ESM.docx]

**Additional file 3.** Effect of motor and epilepsy on survival of *LAMA2*-CMD

| **Variable** | **Survivor**  **(n = 93)** | **Death**  **(n = 23)** | ***P* value** |
| --- | --- | --- | --- |
| Head control, % (n) | 79.6 (74/93) | 56.5 (13/23) | 0.022**^a^** |
| Sitting, % (n) | 90.3 (84/93) | 69.6 (16/23) | 0.010**^a^** |
| Ambulation, % (n) | 18.3 (17/93) | 4.3 (1/23) | 0.119^b^ |
| Epilepsy, % (n) | 4.3 (4/93) | 21.7 (5/23) | 0.015^b^ |

*LAMA2*-CMD: *LAMA2*-related congenital muscular dystrophy.

^a^ Pearson χ^2^ test.

^b^ Fisher’s exact Test.
