# Supplementary material for: Natural history and genetic study of LAMA2-related muscular dystrophy in a large Chinese cohort
Source: Orphanet J Rare Dis. 2021 Jul 19;16:319. doi: 10.1186/s13023-021-01950-x (PMC8287797; doi:10.1186/s13023-021-01950-x)
Supplement: Supplementary file 4 — Additional file 4. Genetical analysis of patients with LAMA2-related muscular dystrophy. [file 13023_2021_1950_MOESM4_ESM.docx]

**Additional file 4.** Genetical analysis of patients with *LAMA2*-related muscular dystrophy

| **Patient** | **Phenotype** | **Exon** | **Domain** | **Nucleotide change** | **Predicted amino acid change** | **ACMG variant-classification** | **Parental derivation** | **Variation type** | **Reported/**  **Novel** |
| --- | --- | --- | --- | --- | --- | --- | --- | --- | --- |
| P1, P2, P3 | *LAMA2*-CMD | 56 | G-like | c.7810C>T | p.R2604* | ACMG: 5 (pathogenic) | P | NS | Reported |
|  |  | 1 | LN | Exon1del |  | ACMG: 5 (pathogenic) | M | CNV | Reported |
| P4 | *LAMA2*-CMD | IVS6 | EGF-like | c.910-1G>T |  | ACMG: 5 (pathogenic) | P | Splicing | Reported |
|  |  | 3-4 | LN | Exon3-4del |  | ACMG: 5 (pathogenic) | De novo | CNV | Reported |
| P5, P14 | *LAMA2*-CMD | 36 | α I | c.5212G>T | p.E1738* | ACMG: 5 (pathogenic) | P | NS | Novel |
|  |  | IVS55 | G-like | c.7750-2A>G |  | ACMG: 5 (pathogenic) | M | Splicing | Reported |
| P6 | *LAMA2*-CMD | 19 | EGF-like | Exon19del |  | ACMG: 5 (pathogenic) | P | CNV | Novel |
|  |  | 23 | EGF-like | c.3294G>A | p.W1098* | ACMG: 5 (pathogenic) | M | NS | Novel |
| P7 | *LAMA2*-CMD | 9 | EGF-like | c.1303C>T | p.R435* | ACMG: 5 (pathogenic) | P | NS | Reported |
|  |  | 14 | IV | c.1942G>T | p.E648* | ACMG: 5 (pathogenic) | M | NS | Reported |
| P8, P56 | *LAMA2*-CMD | 14 | IV | c.2049_2050del | p.R683Sfs*21 | ACMG: 5 (pathogenic) | P | FS | Reported |
|  |  | 5 | LN | Exon5del |  | ACMG: 5 (pathogenic) | M | CNV | Reported |
| P9, P71 | *LAMA2*-CMD | 63 | G-like | c.8906G>C | p.R2969P | ACMG: 3 (VUS) | P | MS | Reported |
|  |  |  | EGF-like | Exon30del |  | ACMG: 5 (pathogenic) | M | CNV | Reported |
| P10, P36 | *LAMA2*-CMD | 18 | EGF-like | c.2462C>T (hom) | p.T821M | ACMG: 3 (VUS) | P, M | MS | Reported |
| P11 | *LAMA2*-CMD | 27 | IV | c.4048C>T | p.R1350* | ACMG: 5 (pathogenic) | P | NS | Reported |
|  |  | IVS38 | α II | c.5563-1G>A |  | ACMG: 5 (pathogenic) | M | Splicing | Novel |
| P12 | *LAMA2*-CMD | 50 | G-like | c.7147C>T | p.R2383* | ACMG: 5 (pathogenic) | P | NS | Reported |
|  |  | 11 | EGF-like | c.1522C>T | p.Q508* | ACMG: 5 (pathogenic) | M | NS | Novel |
| P13 | *LAMA2*-CMD | 14 | IV | c.1976C>A | p.S659* | ACMG: 4 (likely pathogenic) | P | NS | Reported |
|  |  | 57 | G-like | c.7921G>T | p.E2641* | ACMG: 5 (pathogenic) | M | NS | Reported |
| P15, P45 | *LAMA2*-CMD | 29 | EGF-like | c.4031C>A | p.S1434* | ACMG: 5 (pathogenic) | M | NS | Novel |
|  |  | 39 | α II | c.5687dup | p.H1896Qfs*7 | ACMG: 5 (pathogenic) | P | NS | Novel |
| P16 |  | 36 | α I | c.5156_5159del (hom) | p.K1719Rfs*5 | ACMG: 5 (pathogenic) | P, M | FS | Reported |
| P17 | *LAMA2*-CMD | 23 | EGF-like | c.3279C>A | p.C1093* | ACMG: 5 (pathogenic) | P | NS | Novel |
|  |  | 29 | EGF-like | c.4198C>T | p.R1400* | ACMG: 5 (pathogenic) | M | NS | Reported |
| P18, P104 | *LAMA2*-CMD | 14 | IV | c.2049_2050del | p.R683Sfs*21 | ACMG: 5 (pathogenic) | P | FS | Reported |
|  |  | 3 | LN | c.363C>A | p.Y121* | ACMG: 5 (pathogenic) | M | NS | Reported |
| P19 | *LAMA2*-CMD | 42 | α II | c.6050_6053del | p.N2017Tfs*48 | ACMG: 4 (likely pathogenic) | M | FS | Novel |
|  |  | 47 | G-like | c.6584T>C | p.L2195P | ACMG: 3 (VUS) | P | MS | Reported |
| P20 | *LAMA2*-CMD | 37 | α I | c.5290dup | p.E1764Gfs*3 | ACMG: 5 (pathogenic) | P | FS | Reported |
|  |  | 2 | LN | c.250C>T | p.R84* | ACMG: 5 (pathogenic) | M | NS | Reported |
| P21 | *LAMA2*-CMD | 2 | LN | c.283C>T (hom) | p.Q95* | ACMG: 5 (pathogenic) | P, M | NS | Reported |
| P22 | *LAMA2*-CMD | 57 | G-like | c.7921G>T | p.E2641* | ACMG: 5 (pathogenic) | P | NS | Reported |
|  |  | IVS19 | EGF-like | c.2749+2dup |  | ACMG: 4 (likely pathogenic) | NA | Splicing | Reported |
| P23 | *LAMA2*-CMD | IVS49 | G-like | c.6993-2A>G |  | ACMG: 5 (pathogenic) | P | Splicing | Reported |
|  |  | 56 | G-like | c.7898G>C |  | ACMG: 3 (VUS) | M | Predicted splicing | Reported |
| P24 | *LAMA2*-CMD | 23 | EGF-like | c.3283C>T | p.R1095* | ACMG: 5 (pathogenic) | P | NS | Reported |
|  |  | IVS6 | EGF-like | c.910-1G>T |  | ACMG: 5 (pathogenic) | M | Splicing | Reported |
| P25 | *LAMA2*-CMD | 5 | LN | c.817A>T | p.R273* | ACMG: 5 (pathogenic) | P | NS | Reported |
|  |  | 21 | EGF-like | c.3002dup | p.H1001Efs*15 | ACMG: 5 (pathogenic) | M | FS | Novel |
| P26 | *LAMA2*-CMD | 3-4 | LN | Exon3-4del (hom) |  | ACMG: 5 (pathogenic) | P, M | CNV | Reported |
| P27 | *LAMA2*-CMD | 23 | EGF-like | c.3318_3319del | p.C1106* | ACMG: 5 (pathogenic) | P | NS | Novel |
|  |  | 60 | G-like | c.8388A>C | p.E2796D | ACMG: 3 (VUS) | M | MS | Reported |
| P28 | *LAMA2*-CMD | IVS34 | α I | c.4960-2A>G |  | ACMG: 4 (likely pathogenic) | P | Splicing | Reported |
|  |  | 11 | EGF-like | c.1545C>A | p.C515* | ACMG: 4 (likely pathogenic) | M | NS | Novel |
| P29 | *LAMA2*-CMD | 16 | EGF-like | c.2230C>T | p.R744* | ACMG: 5 (pathogenic) | P | NS | Reported |
|  |  | 2 | LN | c.283C>T | p.Q95* | ACMG: 5 (pathogenic) | M | NS | Reported |
| P30 | *LAMA2*-CMD | 4 | LN | Exon4del |  | ACMG: 5 (pathogenic) | P | CNV | Reported |
|  |  | 15 | EGF-like | c.2177G>A | p.C726Y | ACMG: 3 (VUS) | M | MS | Reported |
| P31 | *LAMA2*-CMD | 4 | LN | c.482_485dup | p.E162Dfs*2 | ACMG: 5 (pathogenic) | P | FS | Reported |
|  |  | IVS21 | EGF-like | c.3038-7G>A |  | ACMG: 3 (VUS) | M | Splicing | Reported |
| P32 | *LAMA2*-CMD | 50 | G-like | c.7147C>T | p.R2383* | ACMG: 5 (pathogenic) | P | NS | Reported |
|  |  | 1 | LN | Exon1del |  | ACMG: 5 (pathogenic) | M | CNV | Reported |
| P33 | *LAMA2*-CMD | 65 | G-like | c.9232_9267dup | p.L3078Lfs*39 | ACMG: 4 (likely pathogenic) | P | FS | Novel |
|  |  | IVS6 | LN | c.909+5_909+18del |  | ACMG: 3 (VUS) | M | Splicing | Novel |
| P34 | *LAMA2*-CMD | 57 | G-like | c.8226del | p.T2743Pfs*4 | ACMG: 5 (pathogenic) | P | FS | Novel |
|  |  | 20 | EGF-like | c.2844T>A | p.C948* | ACMG: 5 (pathogenic) | M | NS | Novel |
| P35 | *LAMA2*-CMD | 36 | α I | c.5156_5159del | p.K1719Rfs*5 | ACMG: 5 (pathogenic) | P | FS | Reported |
|  |  | 50 | G-like | c.7147C>T | p.R2383* | ACMG: 5 (pathogenic) | M | NS | Reported |
| P37 | *LAMA2*-CMD | 14 | IV | c.2049_2050del | p.R683Sfs*21 | ACMG: 5 (pathogenic) | P | FS | Reported |
|  |  | 46 | G-like | c.6466C>T | p.R2156* | ACMG: 5 (pathogenic) | M | NS | Reported |
| P38 | *LAMA2*-CMD | 12 | IV | c.1782+2T>G |  | ACMG: 5 (pathogenic) | P | Splicing | Novel |
|  |  | 58 | G-like | c.8217dup | p.P2740Sfs*40 | ACMG: 5 (pathogenic) | M | FS | Reported |
| P39 | *LAMA2*-CMD | 57 | G-like | c.7921G>T | p.E2641* | ACMG: 5 (pathogenic) | P | NS | Reported |
|  |  | 36 | α I | c.5126_5133del | p.A1709Efs*11 | ACMG: 5 (pathogenic) | M | FS | Novel |
| P40, P109 | *LAMA2*-CMD | 17 | EGF-like | c.2447del | p.N816Ifs*9 | ACMG: 5 (pathogenic) | P | FS | Novel |
|  |  | 27 | IV | c.3976C>T | p.R1326* | ACMG: 5 (pathogenic) | M | NS | Reported |
| P41 | *LAMA2*-CMD | IVS2 | LN | c.285_288del | p.R96Tfs*8 | ACMG: 5 (pathogenic) | P | FS | Reported |
|  |  | 27 | IV | c.3955C>T | p.R1319* | ACMG: 5 (pathogenic) | M | NS | Reported |
| P42 | *LAMA2*-CMD |  | G-like | Exon49del |  | ACMG: 5 (pathogenic) | P | CNV | Reported |
|  |  | 50 | G-like | c.7147C>T | p.R2383* | ACMG: 5 (pathogenic) | M | NS | Reported |
| P43 | *LAMA2*-CMD | 36 | α I | c.5156_5159del | p.K1719Rfs*5 | ACMG: 5 (pathogenic) | P | FS | Reported |
|  |  | 14 | IV | c.1900_1903dup | p.S635Nfs*7 | ACMG: 5 (pathogenic) | M | FS | Novel |
| P44 | *LAMA2*-CMD | 4 | LN | Exon4del |  | ACMG: 5 (pathogenic) | P | CNV | Reported |
|  |  | 50 | G-like | c.7147C>T | p.R2383* | ACMG: 5 (pathogenic) | M | NS | Reported |
| P46 | *LAMA2*-CMD | 50 | G-like | c.7147C>T | p.R2383* | ACMG: 5 (pathogenic) | P | NS | Reported |
|  |  | IVS50 | G-like | c.7156-2A>G |  | ACMG: 5 (pathogenic) | M | Splicing | Reported |
| P47 | *LAMA2*-CMD | 4 | LN | c.469T>C | p.S157P | ACMG: 3 (VUS) | P | MS | Reported |
|  |  | 36 | α I | c.5156_5159del | p.K1719Rfs*5 | ACMG: 5 (pathogenic) | M | FS | Reported |
| P48 | *LAMA2*-CMD | 9 | LN | Exon9del |  | ACMG: 4 (likely pathogenic) | P | CNV | Novel |
|  |  | IVS24 | IV | c.3556-1G>A |  | ACMG: 4 (likely pathogenic) | M | Splicing | Novel |
| P49 | *LAMA2*-CMD | 46 | G-like | c.6527_6528del | p.T2176Sfs*4 | ACMG: 5 (pathogenic) | P | FS | Novel |
|  |  | 38 | α I | c.5476C>T | p.R1826* | ACMG: 5 (pathogenic) | M | NS | Reported |
| P50 | *LAMA2*-CMD | 20 | EGF-like | Exon20del |  | ACMG: 5 (pathogenic) | P | CNV | Reported |
|  |  | 46 | G-like | c.6466C>T | p.R2156* | ACMG: 5 (pathogenic) | M | NS | Reported |
| P51, P81 | *LAMA2*-CMD | 52 | G-like | c.7383_7387del | p.D2461Efs*4 | ACMG: 5 (pathogenic) | P | FS | Novel |
|  |  | 1 | LN | c.56dup | p.V20Rfs*30 | ACMG: 5 (pathogenic) | M | FS | Novel |
| P52, P106 | *LAMA2*-CMD | 49-57 | G-like | Exon49-57del |  | ACMG: 5 (pathogenic) | P | CNV | Reported |
|  |  | 46 | G-like | c.6433A>T | p.K2145* | ACMG: 5 (pathogenic) | M | NS | Reported |
| P53 | *LAMA2*-CMD | 31 | EGF-like | c.4522del | p.R1508Gfs*87 | ACMG: 5 (pathogenic) | P | FS | Novel |
|  |  | 14 | IV | c.2055_2057del | p.L685del | ACMG: 3 (VUS) | M | Del | Novel |
| P54 | *LAMA2*-CMD | 15 | EGF-like | c.2206G>T | p.E736* | ACMG: 5 (pathogenic) | P | NS | Novel |
|  |  | 4 | LN | c.463G>T | p.E155* | ACMG: 5 (pathogenic) | M | NS | Reported |
| P55 | *LAMA2*-CMD | IVS25 | IV | c.3735+2_3735+8delinsAAAGAAGGA (hom) |  | ACMG: 4 (likely pathogenic) | P, M | Splicing | Novel |
| P57 | *LAMA2*-CMD | 4 | LN | Exon4del (hom) |  | ACMG: 5 (pathogenic) | P, M | CNV | Reported |
| P58 | *LAMA2*-CMD | IVS21 | EGF-like | c.3038-7G>A |  | ACMG: 3 (VUS) | P | Splicing | Reported |
|  |  | 13-14 | IV | Exon13-14del |  | ACMG: 5 (pathogenic) | M | CNV | Reported |
| P59 | *LAMA2*-CMD | 11 | EGF-like | c.1580G>A | p.C527Y | ACMG: 4 (likely pathogenic) | P | MS | Reported |
|  |  | IVS4 | LN | c.640-1G>C |  | ACMG: 5 (pathogenic) | M | Splicing | Reported |
| P60, P89 | *LAMA2*-CMD | 27 | EGF-like | c.4058G>A |  | ACMG: 4 (likely pathogenic) | P | Predicted splicing | Novel |
|  |  | 46 | G-like | c.6513_6515del | p.V2171del | ACMG: 5 (pathogenic) | M | Del | Reported |
| P61 | *LAMA2*-CMD | 4 | LN | Exon4del |  | ACMG: 5 (pathogenic) | P | CNV | Reported |
|  |  | 50 | G-like | c.7147C>T | p.R2383* | ACMG: 5 (pathogenic) | M | NS | Reported |
| P62 | *LAMA2*-CMD | 10-12 | EGF-like | Exon10-12del |  | ACMG: 5 (pathogenic) | P | CNV | Reported |
|  |  | 64 | G-like | c.9101_9104dup | p.H3035Qfs*5 | ACMG: 5 (pathogenic) | M | FS | Reported |
| P63 | *LAMA2*-CMD | 57 | G-like | c.7991del | p.G2664Vfs*64 | ACMG: 5 (pathogenic) | P | FS | Reported |
|  |  | 26 | IV | c.3904C>T | p.H1302Y | ACMG: 3 (VUS) | M | MS | Novel |
| P64, P105 | *LAMA2*-CMD | 8 | EGF-like | c.1153_1154del | p.T385Cfs*10 | ACMG: 5 (pathogenic) | P | FS | Reported |
|  |  | 59 | G-like | c.8264C>A | p.S2755* | ACMG: 5 (pathogenic) | M | NS | Reported |
| P65 | *LAMA2*-CMD | 18 | EGF-like | c.2526_2529dup | p. C844Tfs*3 | ACMG: 5 (pathogenic) | P | FS | Reported |
|  |  | 49 | G-like | Exon49del |  | ACMG: 5 (pathogenic) | M | CNV | Reported |
| P66 | *LAMA2*-CMD | 37 | α I | c.5290dup | p.E1764Gfs*3 | ACMG: 5 (pathogenic) | P | FS | Reported |
|  |  | 5-8 | LN | Exon5-8dup |  | ACMG: 5 (pathogenic) | M | CNV | Reported |
| P67 | *LAMA2*-CMD | 56 | G-like | c.7888C>T | p.R2630* | ACMG: 5 (pathogenic) | P | NS | Reported |
|  |  | 25 | IV | c.3726del | p.G1243Efs*4 | ACMG: 5 (pathogenic) | M | FS | Novel |
| P68 | *LAMA2*-CMD | 17 | EGF-like | c.2434_2435del | p.N812Yfs*5 | ACMG: 5 (pathogenic) | P | NS | Novel |
|  |  | 57 | G-like | c.7991del | p.G2664Vfs*64 | ACMG: 5 (pathogenic) | M | NS | Reported |
| P69 | *LAMA2*-CMD | 30 | EGF-like | c.4348C>T | p.R1450* | ACMG: 5 (pathogenic) | P | NS | Reported |
|  |  | 27 | IV | c.4048C>T | p.R1350* | ACMG: 5 (pathogenic) | M | NS | Reported |
| P70 | *LAMA2*-CMD | 5 | LN | c.817A>T | p.R273* | ACMG: 5 (pathogenic) | P | NS | Reported |
|  |  | 14 | IV | c.2049_2050del | p.R683Sfs*21 | ACMG: 5 (pathogenic) | M | FS | Reported |
| P72 | *LAMA2*-CMD | 63 | G-like | c.8906G>C | p.R2969P | ACMG: 3 (VUS) | P | MS | Reported |
|  |  | 2-12 | LN | Exon2-12del |  | ACMG: 5 (pathogenic) | M | CNV | Reported |
| P73 | *LAMA2*-CMD | IVS4 | LN | c.640-1G>C (hom) |  | ACMG: 5 (pathogenic) | P, M | Splicing | Reported |
| P74 | *LAMA2*-CMD | 63 | G-like | c.8987del | p. K2996Sfs*2 | ACMG: 5 (pathogenic) | P | FS | Reported |
|  |  | 22 | EGF-like | c.3096C>A | p.C1032* | ACMG: 4 (likely pathogenic) | M | NS | Reported |
| P75 | *LAMA2*-CMD | 57 | G-like | c.7991del | p.G2664Vfs*64 | ACMG: 5 (pathogenic) | P | FS | Reported |
|  |  | 38 | α I | c.5476C>T | p.R1826* | ACMG: 5 (pathogenic) | M | NS | Reported |
| P76 | *LAMA2*-CMD | 59-63 | G-like | Exon59-63del |  | ACMG: 5 (pathogenic) | P | CNV | Reported |
|  |  | 63 | G-like | c.8906G>C | p.R2969P | ACMG: 3 (VUS) | M | MS | Reported |
| P77 | *LAMA2*-CMD | IVS58 | G-like | c.8244+3_8244+6del |  | ACMG: 3 (VUS) | P | Splicing | Reported |
|  |  | 55 | G-like | c.7732C>T | p.R2578* | ACMG: 5 (pathogenic) | M | NS | Reported |
| P78 | *LAMA2*-CMD |  | LN | Exon2-9del |  | ACMG: 5 (pathogenic) | P | CNV | Reported |
|  |  | 47 | G-like | c.6584T>C | p.L2195P | ACMG: 3 (VUS) | M | MS | Reported |
| P79 | *LAMA2*-CMD | 4 | LN | Exon4del |  | ACMG: 5 (pathogenic) | P | CNV | Reported |
|  |  | 19 | EGF-like | c.2565del | p. S856Lfs*32 | ACMG: 5 (pathogenic) | M | FS | Reported |
| P80 | *LAMA2*-CMD | 27 | IV | c.3955C>T (hom) | p.R1319* | ACMG: 5 (pathogenic) | P, M | NS | Reported |
| P82 | *LAMA2*-CMD | 11 | EGF-like | c.1553G>A | p.C518Y | ACMG: 3 (VUS) | P | MS | Reported |
|  |  | 36 | α I | c.5156_5159del | p.K1719Rfs*5 | ACMG: 5 (pathogenic) | M | FS | Reported |
| P83 | *LAMA2*-CMD | 21 | EGF-like | c.2958G>A | p.W986* | ACMG: 5 (pathogenic) | P | NS | Reported |
|  |  | 38 | α I | c.5476C>T | p.R1826* | ACMG: 5 (pathogenic) | M | NS | Reported |
| P84, P114 | *LAMA2*-CMD | 23 | IV | c.3556-13T>A | p.V1186Tfs*4 | ACMG: 5 (pathogenic) | P | FS | Reported |
|  |  | 34 | α I | c.4886del | P1629Qfs*12 | ACMG: 5 (pathogenic) | M | FS | Reported |
| P85 | *LAMA2*-CMD | 10-12 | EGF-like + IV | Exon10-12dup |  | ACMG: 5 (pathogenic) | P | CNV | Reported |
|  |  | 7 | EGF-like | c.928del | p.E310Sfs*27 | ACMG: 5 (pathogenic) | M | FS | Novel |
| P86 | *LAMA2*-CMD | 27 | IV | c.4048C>T | p.R1350* | ACMG: 5 (pathogenic) | P | NS | Reported |
|  |  | 63 | G-like | c.8910_8965del | p.T2921Yfs*2 | ACMG: 5 (pathogenic) | M | FS | Reported |
| P87 | *LAMA2*-CMD | 8 | EGF-like | c.1127del | p.G376Vfs*13 | ACMG: 5 (pathogenic) | M | FS | Reported |
|  |  | 43 | α II | c.6207C>A | p.Y2069* | ACMG: 5 (pathogenic) | P | NS | Reported |
| P88, P90 | *LAMA2*-CMD | IVS2 | LN | c.283+1G>C |  | ACMG: 5 (pathogenic) | M | Splicing | Reported |
|  |  | 56 | G-like | c.7810C>T | p.R2604* | ACMG: 5 (pathogenic) | P | NS | Reported |
| P91 | *LAMA2*-CMD | 16 | EGF-like | c.2230C>T | p.R744* | ACMG: 5 (pathogenic) | P | NS | Reported |
|  |  | 27 | IV | c.4048C>T | p.R1350* | ACMG: 5 (pathogenic) | M | NS | Reported |
| P92 | *LAMA2*-CMD | 27 | IV | c.3931T>G | p.W1311G | ACMG: 5 (pathogenic) | P | MS | Reported |
|  |  | 9 | EGF-like | c.1303C>T | p.R435* | ACMG: 5 (pathogenic) | M | NS | Reported |
| P93 | *LAMA2*-CMD | 4 | LN | Exon4del (hom) |  | ACMG: 5 (pathogenic) | P, M | CNV | Reported |
| P94 | *LAMA2*-CMD | 4 | LN | Exon4del |  | ACMG: 5 (pathogenic) | P | CNV | Reported |
|  |  | 27 | IV | c.3955C>T | p.R1319* | ACMG: 5 (pathogenic) | M | NS | Reported |
| P95, P97 | *LAMA2*-CMD | 2-3 | LN | Exon2-3del |  | ACMG: 5 (pathogenic) | P | CNV | Reported |
|  |  | IVS35 | α I | c.5071+1G>A |  | ACMG: 5 (pathogenic) | M | Splicing | Reported |
| P96 | *LAMA2*-CMD | 50 | G-like | c.7147C>T | p.R2383* | ACMG: 5 (pathogenic) | P | NS | Reported |
|  |  | 46 | G-like | c.6513_6515del | p.V2171del | ACMG: 5 (pathogenic) | M | Del | Reported |
| P98 | *LAMA2*-CMD | 4 | LN | Exon4del |  | ACMG: 5 (pathogenic) | P | CNV | Reported |
|  |  | 57 | G-like | c.7921G>T | p.E2641* | ACMG: 5 (pathogenic) | M | NS | Reported |
| P99 | *LAMA2*-CMD | 2 | LN | c.283C>T | p.Q95* | ACMG: 5 (pathogenic) | P | NS | Reported |
|  |  | 57 | G-like | c.7921G>T | p.E2641* | ACMG: 5 (pathogenic) | M | NS | Reported |
| P100 | *LAMA2*-CMD | IVS27 | IV | c.4058+1G>A |  | ACMG: 5 (pathogenic) | P | Splicing | Reported |
|  |  | 3 | LN | c.329G>A | p.W110* | ACMG: 5 (pathogenic) | M | NS | Reported |
| P101 | *LAMA2*-CMD | 36 | α I | c.5156_5159del | p.K1719Rfs*5 | ACMG: 5 (pathogenic) | P | FS | Reported |
|  |  | 40 | α I | c.5862del | p.K1954Nfs*10 | ACMG: 5 (pathogenic) | M | FS | Novel |
| P102 | *LAMA2*-CMD | 5 | LN | c.817A>T (hom) | p.R273* | ACMG: 5 (pathogenic) | P, M | NS | Reported |
| P103 | *LAMA2*-CMD | 46 | G-like | c.6513_6515del | p.V2171del | ACMG: 5 (pathogenic) | P | Del | Reported |
|  |  | IVS27 | IV | c.4058+1G>A |  | ACMG: 5 (pathogenic) | M | Splicing | Reported |
| P107 | *LAMA2*-CMD | 50 | G-like | c.7147C>T (hom) | p.R2383* | ACMG: 5 (pathogenic) | P, M | NS | Reported |
| P108 | *LAMA2*-CMD | IVS59 | G-like | c.8358-3C>G |  | ACMG: 3 (VUS) | P | Splicing | Reported |
|  |  | 21 | EGF-like | c.2959dup | p.C987Lfs*10 | ACMG: 5 (pathogenic) | M | FS | Reported |
| P110 | *LAMA2*-CMD | 4 | LN | c.482_485dup | p.E162Dfs*2 | ACMG: 5 (pathogenic) | P | FS | Reported |
|  |  | 41-47 | G-like | Exon41-47del |  | ACMG: 5 (pathogenic) | M | CNV | Reported |
| P111 | *LAMA2*-CMD | 59-63 | G-like | Exon59-63del |  | ACMG: 5 (pathogenic) | P | CNV | Reported |
|  |  | 3 | LN | c.363C>G | p.Y121* | ACMG: 5 (pathogenic) | M | NS | Reported |
| P112 | *LAMA2*-CMD | 50 | G-like | c.7147C>T | p.R2383* | ACMG: 5 (pathogenic) | P | NS | Reported |
|  |  | 56 | G-like | c.7810C>T | p.R2604* | ACMG: 5 (pathogenic) | M | NS | Reported |
| P113 | *LAMA2*-CMD | IVS43 | α II | c.6268+2T>C |  | ACMG: 5 (pathogenic) | P | Splicing | Reported |
|  |  | 36 | α I | c.5156_5159del | p.K1719Rfs*5 | ACMG: 5 (pathogenic) | M | FS | Reported |
| P115 | *LAMA2*-CMD | IVS29 | EGF-like | c.4312-3C>G (hom) |  | ACMG: 3 (VUS) | P, M | Splicing | Reported |
| P116 | *LAMA2*-CMD | IVS58 | G-like | c.8245-2A>T |  | ACMG: 5 (pathogenic) | P | Splicing | Reported |
|  |  | IVS29 | EGF-like | c.4311+2T>C |  | ACMG: 5 (pathogenic) | M | Splicing | Novel |
| P117 | LGMDR23 | 4 | LN | c.437C>A | p.S146Y | ACMG: 3 (VUS) | M | MS | Novel |
|  |  | IVS4 | LN | c.640-1G>C |  | ACMG: 5 (pathogenic) | P | Splicing | Reported |
| P118 | LGMDR23 | 65 | G-like | c.9311dup | p.N3104Nfs*39 | ACMG: 5 (pathogenic) | P | FS | Novel |
|  |  | 14 | IV | c.2049_2050del | p.R683Sfs*21 | ACMG: 5 (pathogenic) | M | FS | Reported |
| P119 | LGMDR23 | 62 | G-like | c.8844_8845insAAGGCTC | p.F2949Kfs*21 | ACMG: 5 (pathogenic) | De novo | FS | Novel |
|  |  | 12 | IV | c.1732_1736del | p.L578Afs*30 | ACMG: 5 (pathogenic) | M | FS | Novel |
| P120 | LGMDR23 | 4 | LN | c.437C>A | p.S146Y | ACMG: 3 (VUS) | P | MS | Novel |
|  |  | 27 | IV | c.4048C>T | p.R1350* | ACMG: 5 (pathogenic) | M | NS | Reported |
| P121 | LGMDR23 | 5 | LN | c.830C>T | p.S277L | ACMG: 3 (VUS) | P | MS | Reported |
|  |  | 4 | LN | Exon4del |  | ACMG: 5 (pathogenic) | M | CNV | Reported |
| P122 | LGMDR23 | 56 | G-like | c.7927del | p.R2643Efs*7 | ACMG: 5 (pathogenic) | De novo | FS | Novel |
|  |  | 61 | G-like | c.8815C>T | p.Q2939* | ACMG: 5 (pathogenic) | M | NS | Novel |
| P123 | LGMDR23 | 22 | EGF-like | c.3149del | p.G1050Afs*25 | ACMG: 5 (pathogenic) | P | FS | Reported |
|  |  | 59-63 | G-like | Exon59-63del |  | ACMG: 5 (pathogenic) | M | CNV | Reported |
| P124 | LGMDR23 | 9 | EGF-like | c.1300C>T | p.R434* | ACMG: 5 (pathogenic) | P | NS | Reported |
|  |  | 11 | EGF-like | c.1544G>A | p.C515Y | ACMG: 3 (VUS) | M | MS | Novel |
| P125, P127 | LGMDR23 | 36-65 | α I | Exon36-65del |  | ACMG: 5 (pathogenic) | P | CNV | Reported |
|  |  | 10 | EGF-like | c.1358G>C | p.C453S | ACMG: 3 (VUS) | M | MS | Reported |
| P126 | LGMDR23 | 47 | G-like | c.6634_6645del | p.S2212_G2215del | ACMG: 4 (likely pathogenic) | P | Del | Novel |
|  |  | 3 | LN | c.332A>C | p.Q111P | ACMG: 3 (VUS) | M | MS | Novel |
| P128, P130 | LGMDR23 | 4 | LN | c.437C>T (hom) | p.S146F | ACMG: 3 (VUS) | P, M | MS | Reported |
| P129 | LGMDR23 | 4 | LN | c.443G>A | p.R148Q | ACMG: 3 (VUS) | De novo | MS | Reported |
|  |  | 43 | α II | c.6235del | p.T2079Rfs*24 | ACMG: 5 (pathogenic) | M | FS | Novel |

ACMG: American College of Medical Genetics and Genomics; α I: laminin helical coiled-coil domain I; α II: laminin helical coiled-coil domain II; CNV: copy number variation; FS: frameshift mutation; hom: homozygous; IV: laminin IV type A1 or A2; *LAMA2*-CMD: *LAMA2*-related congenital muscular dystrophy; LGMDR23: limb-girdle muscular dystrophy-23; LN: laminin N-terminal; M: maternal; MS: missense mutation; NS: nonsense mutation; P: paternal; Splicing: splicing mutation; VUS: variant of uncertain significance.
