# Supplementary material for: Natural history and genetic study of LAMA2-related muscular dystrophy in a large Chinese cohort
Source: Orphanet J Rare Dis. 2021 Jul 19;16:319. doi: 10.1186/s13023-021-01950-x (PMC8287797; doi:10.1186/s13023-021-01950-x)
Supplement: Supplementary file 5 — Additional file 5. Pathogenicity analysis of 17 missense variants. [file 13023_2021_1950_MOESM5_ESM.docx]

**Additional file 5.** Pathogenicity analysis of 17 missense variants

| **No.** | **Nucleotide change** | **Predicted amino acid change** | **Reported/ Novel** | **ACMG variant-classification** | **CADD (Score)** | **PolyPhen-2 HumVar (Score)** | **SIFT PROVEAN Protein (Score)** | **Mutation Taster (Probability)** |
| --- | --- | --- | --- | --- | --- | --- | --- | --- |
| 1 | c.332A>C | p.Q111P | Novel | ACMG: 3 (VUS) | Damaging (27.5) | Probably damaging (0.999) | Deleterious (-5.415) | Disease causing (0.9999999) |
| 2 | c.437C>A | p.S146Y | Novel | ACMG: 3 (VUS) | Damaging (27.4) | Probably damaging (1.000) | Deleterious (-4.748) | Disease causing (0.9999934) |
| 3 | c.1544G>A | p.C515Y | Novel | ACMG: 3 (VUS) | Damaging (29.9) | Probably damaging (1.000) | Deleterious (-9.592) | Disease causing (0.9999999) |
| 4 | c.3904C>T | p.H1302Y | Novel | ACMG: 3 (VUS) | Damaging (25.7) | Probably damaging (0.999) | Neutral (-2.030) | Disease causing (0.9999996) |
| 5 | c.437C>T | p.S146F | Reported | ACMG: 3 (VUS) | Damaging (28.1) | Probably damaging (1.000) | Deleterious (-4.748) | Disease causing (0.9999965) |
| 6 | c.443G>A | p.R148Q | Reported | ACMG: 3 (VUS) | Damaging (34.0) | Probably damaging (1.000) | Deleterious (-3.744) | Disease causing (0.9999835) |
| 7 | c.469T>C | p.S157P | Reported | ACMG: 3 (VUS) | Damaging (27.7) | Probably damaging (1.000) | Deleterious (-4.680) | Disease causing (0.9999997) |
| 8 | c.830C>T | p.S277L | Reported | ACMG: 3 (VUS) | Damaging (34.0) | Probably damaging (1.000) | Deleterious (-5.298) | Disease causing (0.9999999) |
| 9 | c.1358G>C | p.C453S | Reported | ACMG: 3 (VUS) | Damaging (28.2) | Probably damaging (1.000) | Deleterious (-8.831) | Disease causing (0.9999997) |
| 10 | c.1553G>A | p.C518Y | Reported | ACMG: 3 (VUS) | Damaging (31.0) | Probably damaging (0.999) | Deleterious (-9.592) | Disease causing (0.9999999) |
| 11 | c.1580G>A | p.C527Y | Reported | ACMG: 4 (likely pathogenic) | Damaging (32.0) | Probably damaging (0.999) | Deleterious (-9.458) | Disease causing (0.9999999) |
| 12 | c.2177G>A | p.C726Y | Reported | ACMG: 3 (VUS) | Damaging (26.2) | Probably damaging (1.000) | Deleterious (-10.029) | Disease causing (0.9999999) |
| 13 | c.2462C>T | p.T821M | Reported | ACMG: 3 (VUS) | Damaging (26.6) | Probably damaging (1.000) | Deleterious (-4.515) | Disease causing (0.9999490) |
| 14 | c.3931T>G | p.W1311G | Reported | ACMG: 5 (pathogenic) | Damaging (27.2) | Probably damaging (1.000) | Deleterious (-12.084) | Disease causing (0.9999998) |
| 15 | c.6584T>C | p.L2195P | Reported | ACMG: 3 (VUS) | Damaging (32.0) | Probably damaging (1.000) | Deleterious (-6.024) | Disease causing (0.9999999) |
| 16 | c.8388A>C | p.E2796D | Reported | ACMG: 3 (VUS) | Tolerable (13.95) | Benign (0.393) | Neutral (-0.884) | Polymorphism (0.6907963) |
| 17 | c.8906G>C | p.R2969P | Reported | ACMG: 3 (VUS) | Damaging (34.0) | Probably damaging (1.000) | Deleterious (-5.715) | Disease causing (0.9999999) |
