# Supplementary material for: Natural history and genetic study of LAMA2-related muscular dystrophy in a large Chinese cohort
Source: Orphanet J Rare Dis. 2021 Jul 19;16:319. doi: 10.1186/s13023-021-01950-x (PMC8287797; doi:10.1186/s13023-021-01950-x)
Supplement: Supplementary file 6 — Additional file 6. Genotype-phenotype correlations in patients with LAMA2-related muscular dystrophy. [file 13023_2021_1950_MOESM6_ESM.docx]

**Additional file 6.** Genotype-phenotype correlations in patients with *LAMA2*-related muscular dystrophy

| **Variable** | **Survivor**  **(*n* = 106)** | **D****eath**  **(*n* = 24)** | ***LAMA2*-CMD**  **(*n* = 116)** | **LGMDR23**  **(*n* = 14)** |
| --- | --- | --- | --- | --- |
| NS, *n* (%) | 54 (50.9) | 15 (62.5) | 66 (56.9) | 3 (21.4) |
| FS, *n* (%) | 43 (40.6) | 7 (29.2) | 45 (38.8) | 5 (35.7) |
| CNV, *n* (%) | 28 (26.4) | 12 (50.0) | 36 (31.0) | 4 (26.6) |
| MS, *n* (%) | 21 (19.8) | 4 (16.7) | 15 (12.9) | 10 (71.4) |
| Splicing, *n* (%) | 28 (26.4) | 3 (12.5) | 30 (25.9) | 1 (7.1) |
| FS + NS, *n* (%) | 17 (16.03) | 4 (16.7) | 20 (17.2) | 1 (7.1) |
| NS + NS, *n* (%) | 15 (14.2) | 3 (12.5) | 18 (15.5) | 0 (0.0) |
| FS + FS, *n* (%) | 8 (7.5) | 1 (4.2) | 7 (6.0) | 2 (14.3) |
| NS + CNV, *n* (%) | 8 (7.5) | 6 (2.5) | 14 (12.1) | 0 (0.0) |
| NS + splicing, *n* (%) | 9 (8.5) | 2 (8.3) | 11 (9.5) | 0 (0.0) |
| FS + CNV, *n* (%) | 8 (7.5) | 1 (4.2) | 8 (6.9) | 1 (7.1) |
| FS + splicing, *n* (%) | 4 (3.8) | 1 (4.2) | 5 (4.3) | 0 (0.0) |

CNV: copy number variation; FS: frameshift; *LAMA2*-CMD: *LAMA2*-related congenital muscular dystrophy; LGMDR23: limb-girdle muscular dystrophy-23; MS: missense variants; NS: nonsense; splicing: splicing variants.
